# CO_2_-enhanced TADF of an ultra-stable Cu(i) cluster *via* guest–host π–π interaction†

**DOI:** 10.1039/d4sc07949c

**Published:** 2025-03-10

**Authors:** Hong-Jin Zhang, Zong-Ren Chen, Ji-Tong Xu, Jia-Wen Ye, Ling Chen, Xiao-Ming Chen

**Affiliations:** a Jiangmen Key Laboratory of Synthetic Chemistry and Cleaner Production, School of Environmental and Chemical Engineering, Wuyi University Jiangmen Guangdong 529000 PR China wyuchemyjw@126.com wyuchemcling@126.com; b Jiangmen Key Laboratory of Synthetic Chemistry and Cleaner Production, College of Textile Science and Engineering, Wuyi University Jiangmen Guangdong 529000 PR China; c MOE Key Laboratory of Bioinorganic and Synthetic Chemistry, School of Chemistry, IGCME, Sun Yat-Sen University Guangzhou 510275 PR China

## Abstract

Efficient and reversible luminescence detection for CO_2_ without solvent assistance is of great significance but remains challenging to achieve, due to the lack of efficient interaction between CO_2_ molecules and the host emitting center. Benefiting from the abundant host–guest interactions, metal clusters provide a platform for detecting small molecules. However, the insufficient chemical stability of most metal clusters limits their practical applications. Here, we report a hydrophobic Cu(i) cluster (denoted as CuIDPO) with one-dimensional channels. Notably, it displays exceptional chemical stability in both acidic and alkaline aqueous solutions (pH = 1–14). More importantly, CuIDPO shows remarkable CO_2_-induced luminescence enhancement (up to 385% under 1 bar CO_2_), which can be applied to analyze CO_2_ content (LOD = 7.7 mbar). Crystallographic analysis and theoretical calculations suggest the mechanism of CO_2_-locking rotation of the phenyl groups in the Cu(i) cluster through guest–host π–π interaction, which is quite unique when compared to the known acid–base neutralization and framework flexibility adjustment mechanisms. Such luminescence CO_2_ sensing shows advantages like ultrafast response and good reversibility. Additionally, CuIDPO-loaded membranes were fabricated for spatially resolved 2D visual detection.

## Abstract 2

A novel CO_2_ locking mechanism for fluorescence sensing is proposed. Specifically, CO_2_ restricts the rotation of phenyl groups in CuIDPO, suppressing non-radiative decay pathways and thereby enhancing fluorescence emission.

## Graphical abstract

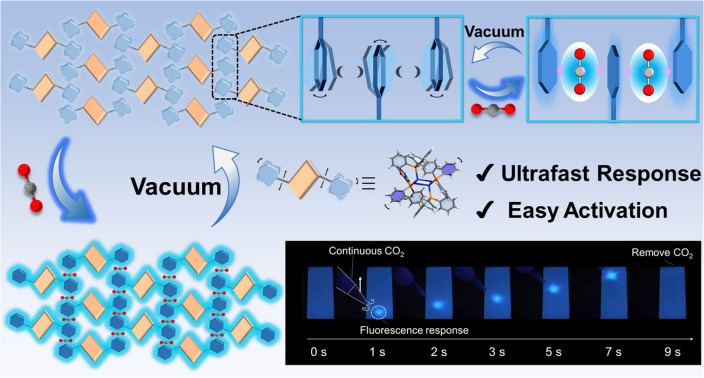

## Introduction

The detection of CO_2_ is crucial in various fields like agriculture,^1^ biology,^2^ carbon emissions^3–5^ and so on. Traditional methods primarily rely on electrochemical technology,^6,7^ Fourier-transform infrared (FT-IR) spectroscopy,^8^ mass spectrometry techniques,^9,10^ and luminescence analysis methods. Notably, luminescence detection offers the advantages of non-electrical connection, convenient operation, high sensitivity and two-dimensional visual detection.^11,12^ Reported luminescence CO_2_ detecting cases usually proceed in solutions, based on acid–base neutralization reactions, as CO_2_ causes weak acidity.^13–16^ However, such a process usually requires the assistance of solvents and is hard to reverse, which is not favourable for the regeneration of optical probes after absorbing CO_2_. In the meantime, there are also several reports that explore CO_2_ sensing performance in porous solid-state materials, for example, flexible metal–organic frameworks (MOFs), *via* employing the flexible changes of the framework caused by the adsorption/desorption of CO_2_ to achieve luminescence changes. These materials no longer require the assistance of solvents, and the activation of fluorescence probes is further facilitated. However, their sensitivity and device fabrication still need enhancement, because of the lack of proper interaction between the CO_2_ molecule and the host emitting center, as well as a simple synthesis method.^17–19^ Therefore, the exploration of efficient luminescent probes for CO_2_ with new sensing mechanisms is an essential and attractive topic.

Clusters of d^10^ metal ions like Cu(i) and Ag(i) possess excellent luminescence stimulus-responsive properties due to the rich transition modes,^20^ making them highly promising for sensing temperature,^21^ pressure,^22^ gas,^23^ and solvent molecules.^24^ The capping effect of ligands can effectively enhance the water stability of d^10^ metal clusters.^25–27^ However, due to the relatively weak coordination bonding between the ligands and d^10^ metal ions, these clusters are more susceptible to most acids and bases compared with other coordination compounds.^28^ Although the increasing hydrophobicity of metal clusters can enhance their resistance to acids and bases, there are still very few related reports.^29–31^

Here, we report a new discrete Cu(i) cluster with one-dimensional pores. This solid compound possesses excellent stability in both acidic and alkaline aqueous solutions (pH = 1–14), due to its strong hydrophobicity. Additionally, it displays blue thermally activated delayed fluorescence (TADF), which can be efficiently enhanced by CO_2_ (by 385% under 1 bar CO_2_). Such a characteristic is then further used for efficient luminescence CO_2_ detection with a fast response and good reversibility, as well as high sensitivity and a low limit of detection (LOD = 7.7 mbar). Single-crystal X-ray diffraction (SCXRD) analysis and theoretical calculations clearly suggest that a guest–host π–π interaction is formed between CO_2_ and the phenyl groups in the Cu(i) cluster, which restricts the host molecular rotation and reduces non-radiative transitions, thus generating unique CO_2_-enhanced luminescence. This unique mechanism provides a new direction for the design of sensitive optical probes for detecting CO_2_. Finally, this metal cluster is also successfully loaded into membranes to achieve spatially resolved two-dimensional visual detection.

## Results and discussion

**Fig. 1:**
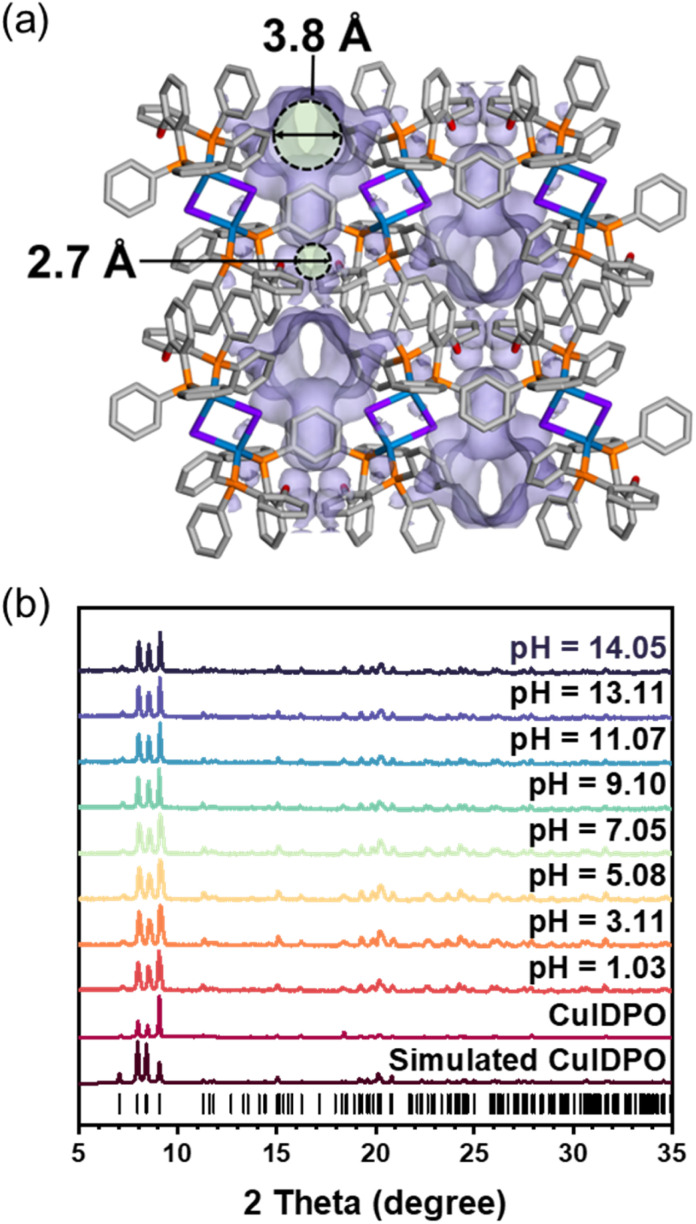
(a) Crystal structure and void surface of CuIDPO viewed along the *b*-axis. Hydrogen atoms are omitted for clarity. Color codes: Cu, blue; I, purple; P, orange; C, grey; O, red. (b) PXRD patterns of CuIDPO after immersion in aqueous solutions with different pH values.

The Cu(i) cluster compound, [Cu_2_I_2_(DPO)_2_]·4CH_2_Cl_2_ (DPO = bis(2-diphenylphosphinophenyl)ether), denoted as CuIDPO·CH_2_Cl_2_, was obtained by the reaction of the DPO ligand and CuI in CH_2_Cl_2_ solvent (Fig. S1†). SCXRD data show that CuIDPO·CH_2_Cl_2_ crystallizes in the monoclinic *P*2_1_/*c* space group with the asymmetric unit consisting of one CuI, one DPO ligand and two CH_2_Cl_2_ guest molecules (Table S1 and Fig. S2†). The discrete [Cu_2_I_2_] cluster is formed by two tetrahedrally coordinated Cu(i) ions, which are bridged by two I^−^ ions and further chelated by DPO ligands. These clusters stack with each other through C–H⋯π interactions in the lattice (Fig. S3†), while CH_2_Cl_2_ molecules occupy hydrophobic channels through van der Waals interactions (Fig. S4†). Remarkably, CH_2_Cl_2_ guests can be completely removed from CuIDPO·CH_2_Cl_2_ through a single-crystal to single-crystal (SC–SC) transformation under gentle operation like vacuuming at room temperature, yielding a guest-free structure (denoted as CuIDPO, Fig. S5†). After removing the CH_2_Cl_2_ molecules, although the cell volume decreases from 3711.92(10) to 3235.0(8) Å^3^ (Table S1†), CuIDPO still retains the one-dimensional (1D) wavy pores with 4.7% porosity (Fig. S6†), generating two distinct micropores (cavity A: 3.8 Å; cavity B: 2.7 Å; Fig. 1a). Additionally, no significant change in the configuration of the DPO ligand is found after guest desorption, whereas a decrease in the intramolecular Cu–Cu distance from 3.55 Å (CuIDPO·CH_2_Cl_2_) to 3.31 Å (CuIDPO) is observed (Fig. S7†).

Commonly, d^10^ metal clusters are highly susceptible to acids and bases, which usually lead to the collapse of cluster structures.^32^ Exceptionally, after immersion in aqueous solutions with a wide pH range (1.03–14.05) for 1 month, CuIDPO can still retain the original crystalline phase (Fig. 1b, S8a and b†), due to its high hydrophobicity with a contact angle of 124.75° (Fig. S8c†). In addition, CuIDPO can retain the original crystalline phase even after long-term exposure (for 8 months) to ambient air conditions (Fig. S9†), further proving its chemical stability. This feature corresponds to facing-out petal-like aromatic rings of DPO that encapsulate the [Cu_2_I_2_] cluster (Fig. S10†). Moreover, as shown in Fig. S11,† the crystallographic planes (100), (010) and (001) are all hydrophobic, so [Cu_2_I_2_] clusters are well shielded and effectively stabilized in the framework when immersed in acidic and basic aqueous solutions. In addition, the relatively hydrophobic pore surface in CuIDPO is also beneficial for high stability (Fig. S12†). Thermogravimetric analysis (TGA) shows that after exposure to humid N_2_ (Fig. S13†), the weight change of CuIDPO is minimal (less than 0.25%), which further demonstrates the hydrophobic nature of the CuIDPO channels. Moreover, TGA of CuIDPO shows that the structure does not collapse till 340 °C (Fig. S14†), suggesting its high thermostability.

**Fig. 2:**
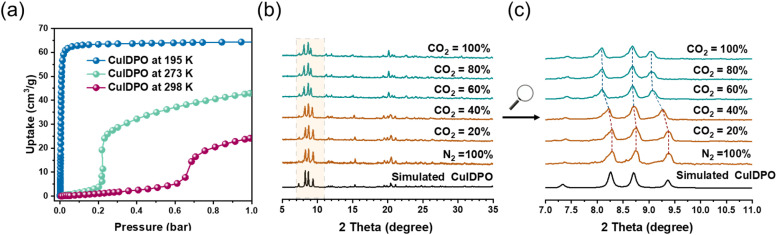
(a) CO_2_ sorption isotherms of CuIDPO at 195, 273 and 298 K. (b) PXRD patterns of CuIDPO in CO_2_/N_2_ mixtures of different ratios (v/v). (c) Enlarged patterns of (a) for 2*θ* = 7–11°.

CuIDPO shows negligible N_2_ adsorption at 77 K (0.125 mmol g^−1^ at *P*/*P*_0_ = 0.90, Fig. S15†), attributed to the extremely narrow channel apertures and quasi-discrete pores. In contrast, CO_2_ adsorption follows a type-I isotherm at 195 K (Fig. 2a). The experimental saturated CO_2_ uptake is 2.92 mmol g^−1^ at *P*/*P*_0_ = 0.94, which is slightly lower than the empirically calculated value (3.25 mmol g^−1^) with a Langmuir surface area of 374.95 m^2^ g^−1^ at 195 K (Fig. 2a and S16†). Additionally, at 273 K and 298 K, the CO_2_ sorption isotherms exhibit an S-shaped profile, revealing significant breathing or gate-opening behaviour with the gate-opening pressures (*P*_go_) of 0.2 and 0.6 bar at 273 and 298 K, respectively. Specifically, at 298 K, the saturated CO_2_ uptake is 1.06 mmol g^−1^, corresponding to 1.55 CO_2_ molecules per unit cell. For 273 K, these values increase to 1.92 mmol g^−1^ and 2.80 CO_2_ molecules per unit cell, respectively. To further elucidate the gate-opening process of CuIDPO, PXRD patterns in 1 bar gas mixtures with different ratios (v/v) of CO_2_ and N_2_ were recorded (Fig. 2b). The PXRD patterns remain unchanged after exposure to 0–40% CO_2_ and are consistent with the simulated pattern of CuIDPO, illustrating that no gate-opening effect occurs at low CO_2_ content. However, upon further increasing the CO_2_ content (≥60%), a variation can be observed in the PXRD pattern. As shown in Fig. 2c, the peak at 9.4° shifts to 9.0°, indicating that a new phase has emerged.

The photophysical properties of CuIDPO were subsequently investigated. An absorption band at 200–400 nm is found in the UV-vis adsorption spectrum of CuIDPO (Fig. S17†), corresponding to the white appearance of the powder sample under ambient light. Density functional theory (DFT) and time-dependent density functional theory (TDDFT) calculations show that the highest energy absorption (S_0_ → S_2_) can be assigned to the mixed metal/halogen to ligand charge transfer (M/XLCT, Fig. S18†). Moreover, as seen in the crystal structure of CuIDPO, the Cu⋯Cu distance is 3.38 Å (much larger than 3 Å), indicating no significant Cu⋯Cu interaction (Fig. S7 and Table S3†).^33^ Under excitation of 365 nm LED light, the maximum emission wavelength (*λ*_em_) of CuIDPO is located at 432 nm with a lifetime of 0.47 μs in air *via* T_1_ → S_0_ transitions from M/XLCT states (Fig. S19†). Temperature-dependent (80 to 360 K) emission spectra show that, with the increase of temperature, the emission of CuIDPO decreases in intensity and exhibits a blue shift (Fig. S20†). Such thermochromic behaviour resembles those of highly emissive solid-state Cu(i) complexes, which is ascribed to the TADF mechanism.^33,34^ In the analysis of the decay curve for luminescence lifetime in a vacuum, by fitting the long-lived component to eqn S1,† the results of Δ*E*(S_1_ − T_1_) = 0.1076 eV (<0.2 eV), indicating a low energy gap for reverse intersystem crossing (RISC), *τ*(S_1_) = 52 ns and *τ*(T_1_) = 224 μs can be obtained (Fig. S21†). Such narrow Δ*E*(S_1_ − T_1_) is also confirmed by TDDFT calculations (Fig. S22†).

**Fig. 3:**
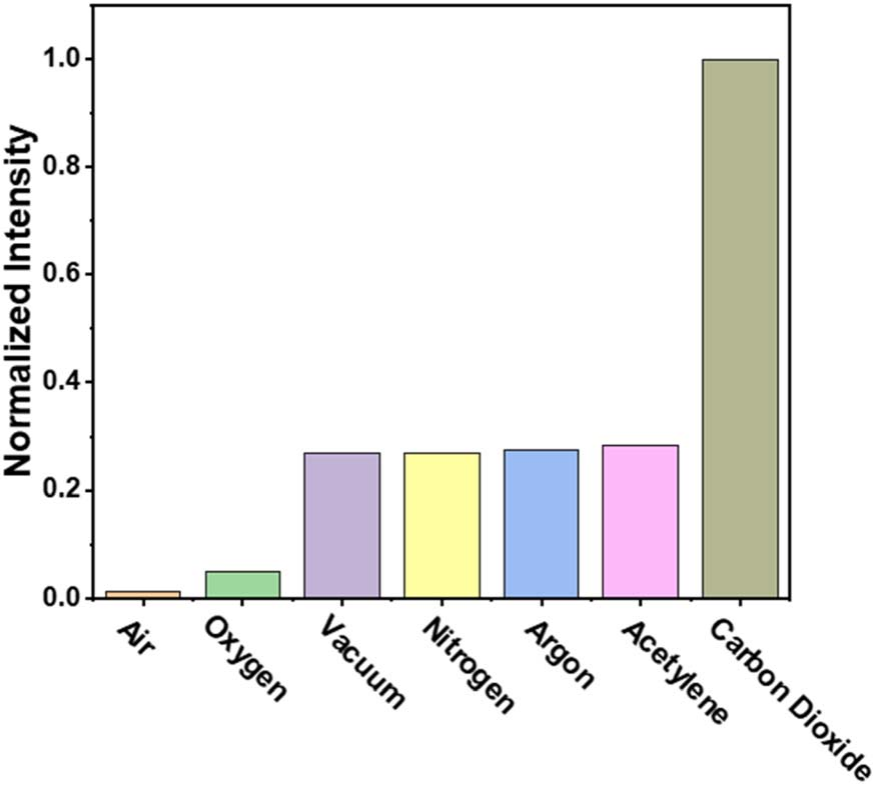
Emission intensities of CuIDPO in different gases and a vacuum, excited with a 365 nm LED and detected at 432 nm.

Interestingly, under 1 bar CO_2_, an obvious enhancement of luminescence intensity is observed for CuIDPO, which is about 5 times that collected in a vacuum (Fig. 3 and S23†). Additionally, other common gases, such as O_2_, air, N_2_ and Ar, cannot enhance the emission of CuIDPO, demonstrating its high selectivity for CO_2_ sensing (Fig. 3 and S23†). Even C_2_H_2_, which is a linear molecule with a high quadrupole moment, cannot affect its fluorescence (Fig. 3 and S23†). As this CO_2_ light-up emission is quite unique in reported solid complexes, the corresponding study is performed in detail next.

**Fig. 4:**
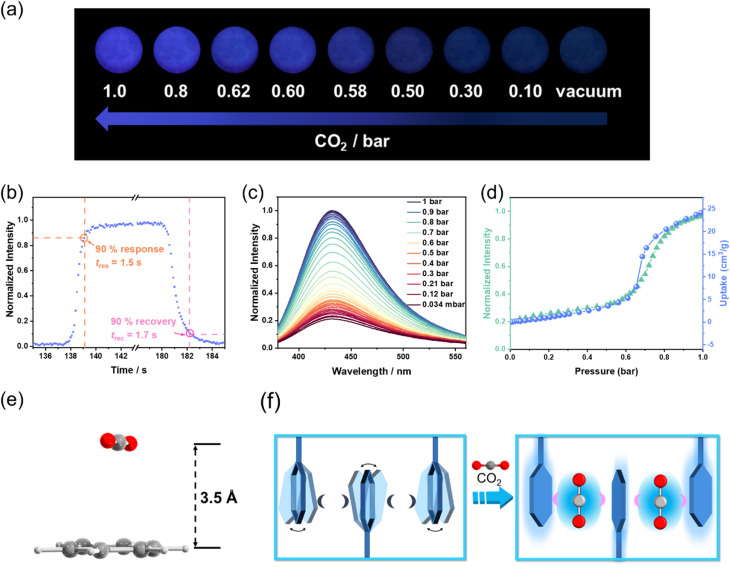
(a) Photographs of CuIDPO at different CO_2_ pressures and room temperature, excited with a 365 nm UV lamp. (b) Enlarged view of one cycle of the kinetic scan of CuIDPO under alternating vacuum and CO_2_ conditions, excited at 365 nm and detected at 432 nm. (c) Emission spectra of CuIDPO at different CO_2_ pressures, excited at 365 nm. (d) Comparison between the luminescence intensity (excited at 365 nm and detected at 432 nm) and CO_2_ uptakes (298 K) of CuIDPO at different CO_2_ pressures. (e) The π–π interaction between CO_2_ and the phenyl ring of CuIDPO·CO_2_. (f) Schematic diagram of the CO_2_ sensing mechanism. The pink and dark crescents represent the presence of and the lack of interaction between the phenyl groups and the guest molecules, respectively.

Upon alternating exposure to CO_2_ and vacuum, CuIDPO exhibits reversible switching between bright blue emission (“on” state) and near-quenched emission (“off” state) under 365 nm illumination (Fig. 4a). No obvious decay of the intensity appears even after 10 alternating cycles, indicating its high optical stability (Fig. S24†). Moreover, the CO_2_ sensing shows very rapid responding/recovering speeds. Specifically, the response time (*t*_res_) and the recovery time (*t*_rec_) are defined as the time when the luminescence intensity of the detecting intensity changes more than 90%. The “measured” *t*_res_ and *t*_rec_ are 1.5 and 1.7 s, by switching the atmosphere between CO_2_ and vacuum, respectively (Fig. 4b). Actually, the visual color transition appears instantaneous. When gradually increasing the CO_2_ pressure from 0 to 1 bar at room temperature, a monotonic luminescence intensity enhancement can be observed across the entire process (excited at 365 nm, Fig. 4c). In particular, the pressure–intensity curve is almost coincided with the sorption isotherms of CuIDPO for CO_2_ at 298 K (Fig. 4d), confirming a direct correlation between CO_2_ uptake and emission enhancement. Moreover, the luminescence intensities of CuIDPO exhibit a good linear relationship with the concentration of CO_2_ in the range of 0–0.2 bar. Hence, the LOD concentration for CO_2_ can be calculated to be 7.7 mbar based on eqn (S2) (Fig. S25).† Additionally, CuIDPO can retain the original crystalline (CuIDPO) phase after CO_2_ absorption and CO_2_ response experiments (Fig. S26†), further confirming its high stability. Except for the luminescence intensity enhancement, CO_2_ also helps to increase the lifetime of CuIDPO, which can be increased to 13.59 μs when under 1 bar CO_2_ (Fig. S27†). Moreover, even after soaking CuIDPO in aqueous solutions with pH = 1 (HCl solution) and pH = 14 (NaOH solution), respectively, for as long as one week, CuIDPO still retains its fluorescence sensing ability for CO_2_ (Fig. S28 and S29†), further demonstrating the chemical stability of CuIDPO.

To further confirm the mechanism of CO_2_-enhanced emission, the interaction between CO_2_ and CuIDPO was investigated. SCXRD data of CuIDPO were collected under 1 bar CO_2_. Crystallographic analysis reveals that, at 291 K, CO_2_ molecules are absorbed at cavity A, which is highly disordered (Fig. S30†). After cooling to 150 K, the CO_2_ molecules became ordered (Fig. S31†), and this CO_2_-absorbed structure is denoted as CuIDPO·CO_2_. The PXRD pattern of CuIDPO under 1 bar CO_2_ is consistent with the simulated PXRD pattern of CuIDPO·CO_2_ (Fig. S32†). CuIDPO·CO_2_ crystallizes in the monoclinic *P*2_1_/*n* space group, and the cell volume (3381.77(6) Å^3^) expands, compared with that of CuIDPO (150 K, 3235.0(8) Å^3^, Table S2†). Though the Cu⋯Cu distance extends from 3.31 Å (CuIDPO at 150 K) to 3.51 Å (CuIDPO·CO_2_ at 150 K, ≫3 Å), it was still too long to generate effective Cu⋯Cu interaction (Fig. S33 and Table S3†). Interestingly, CO_2_ is very close (3.5 Å) to one of the phenyl rings in the framework, which is close enough to form a π–π interaction (Fig. 4e). This π–π interaction locks the rotation of the free phenyl ring, significantly reducing non-radiative transitions and enhancing luminescence (Fig. 4f). DFT calculations show that the binding energy between CO_2_ and this phenyl ring is about −13.92 kJ mol^−1^ (Table S4†), which is close to the common energy level of reported CO_2_–phenyl π–π interaction.^35^ Moreover, when CuIDPO absorbs CO_2_, the *λ*_em_ of CuIDPO remains unchanged, proving that the configuration of its luminescent center has remained essentially unchanged (Fig. 4c). This fact excludes the formation of exciplexes and the process of intramolecular charge transfer, which require the influence to the transition of the molecular orbitals in CuIDPO, attributed to the nonpolar nature of CO_2_. Therefore, the luminescence enhancement solely originates from the CO_2_-caused restriction of the molecular rotation of phenyl groups in CuIDPO (Fig. 4f), similar to the restriction of intramolecular motion (RIM) effect in the aggregation-induced emission luminogens (AIEgens). Similar to CO_2_, the incorporation of CH_3_CN into the framework induces a comparable luminescence enhancement, suggesting a similar host–guest interaction mechanism (Fig. S34 and S35†). The crystal structure of the CH_3_CN-absorbed compound (CuIDPO·CH_3_CN) shows that C–H⋯π interactions between CH_3_CN and the metal cluster are formed with a binding energy of −16.02 kJ mol^−1^ (Fig. S36 and Table S4†), further illustrating the importance of RIM.

**Fig. 5:**
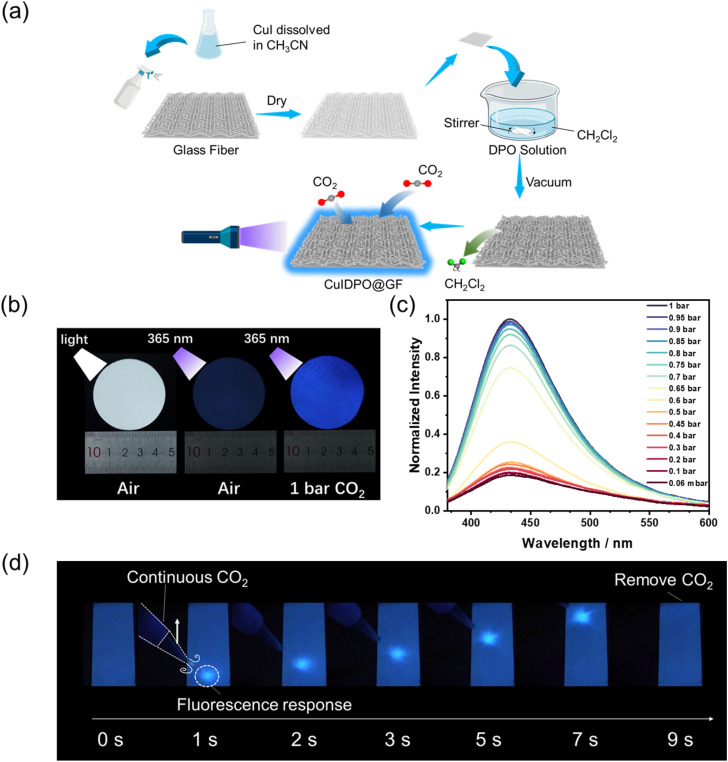
(a) Schematic illustration of *in situ* synthesis of CuIDPO@GF. Color codes for CO_2_ and CH_2_Cl_2_: C, grey; O, red; Cl, green; H, white. (b) Photographs of CuIDPO@GF, excited by daylight and 365 nm UV light, respectively, in air and under 1 bar CO_2_. (c) Emission spectra of CuIDPO@GF at different CO_2_ pressures, excited with a 365 nm UV LED. (d) Luminescence photographs of CuIDPO@GF under CO_2_ purged using a glass needle, excited with a 365 nm UV LED.

To further increase the portability and visualizability of CuIDPO in optical CO_2_ sensing, membrane sensors based on glass fiber (GF) paper were fabricated. First, a CH_3_CN solution of CuI was sprayed onto GF paper, allowing the GF paper to fully absorb CuI. After drying, it was soaked in a CH_2_Cl_2_ solution of DPO to conduct a reaction between CuI and DPO within the GF paper, resulting in the formation of CuIDPO@GF (Fig. 5a and b). The PXRD pattern shows that CuIDPO on the GF paper maintains its crystalline phase (Fig. S37†). The scanning electron microscope (SEM) photographs show that the CuIDPO particles adhere to the GF paper well (Fig. S38†). CuIDPO@GF exhibit not only similar photophysical properties to CuIDPO, but also high sensitivity and fast response to CO_2_ (Fig. 5c, d and S39†). This membrane achieves two-dimensional visual detection of CO_2_. As shown in Fig. 5d and Video S1,† when exposed to a CO_2_ flow, a bright spot is immediately observed in the corresponding site of CuIDPO@GF. However, the spot turns dark as soon as the CO_2_ flow is removed, indicating a great possibility for displaying the pressure of CO_2_ in two dimensions. To test the responsiveness of CuIDPO@GF in high humidity (82% RH) environments, we bubbled CO_2_ through water before it came into contact with CuIDPO@GF (Fig. S40†). After 10 cycles, CuIDPO@GF retains its responsiveness without any decay (Fig. S41†). Delightfully, CuIDPO@GF retains a rapid response time even under high humidity (82% RH) conditions (Fig. S42†). These facts indicate that CuIDPO@GF has great potential to operate in more complex environments.

## Conclusions

A Cu(i) cluster with blue TADF was synthesized, which exhibits excellent stability in acidic and basic environments. Its emission can be selectively enhanced by CO_2_, based on which a new CO_2_ sensor with rapid response, high selectivity and good reversibility is developed. Experimental and theoretic studies indicate that the luminescence enhancement phenomenon caused by CO_2_ can be attributed to a new response mechanism, that is, strong π–π interaction between CO_2_ and the phenyl groups of CuIDPO. Such interaction restricts the molecular rotation of CuIDPO, resulting in the reduction of non-radiative transitions, thus enhancing the luminescence intensity. Furthermore, to achieve spatially resolved two-dimensional visual detection, CuIDPO was successfully loaded onto GF paper to form membrane sensors.

## Data availability

All data have been included in the main text and ESI.†

## Author contributions

Jia-Wen Ye designed the research. Hong-Jin Zhang performed syntheses and most of the measurements. Zong-Ren Chen and Ji-Tong Xu assisted with crystallographic data. Jia-Wen Ye, Ling Chen and Xiao-Ming Chen analysed data and wrote the manuscript.

## Conflicts of interest

The authors declare that they have no conflict of interest.

## Supplementary Material

SC-016-D4SC07949C-s001

SC-016-D4SC07949C-s002

SC-016-D4SC07949C-s003
