# Supplementary material for: CO2-enhanced TADF of an ultra-stable Cu(i) cluster via guest–host π–π interaction
Source: Chem Sci. 2025 Mar 10;16(16):6872–8. doi: 10.1039/d4sc07949c (PMC11920900; doi:10.1039/d4sc07949c)
Supplement: SC-016-D4SC07949C-s001 [file SC-016-D4SC07949C-s001.pdf]

Supplementary Information (SI) for Chemical Science.
This journal is © The Royal Society of Chemistry 2025

Electronic Supplementary Material (ESI) for Chemical Science
This journal is © The Royal Society of Chemistry 2025

CO_2-Enhanced TADF of an Ultra-Stable Cu(I) Cluster via Guest-Host π–π Interaction

Hong-Jin Zhang,[a] Zong-Ren Chen,[a] Ji-Tong Xu,[b] Jia-Wen Ye,*,[a] Ling Chen*,[a] and Xiao-Ming Chen.[c]

[a] Jiangmen Key Laboratory of Synthetic Chemistry and Cleaner Production, School of Environmental and Chemical Engineering, Wuyi University, Jiangmen, Guangdong 529000, PR China.

[b] School of Textile Science and Engineering, Wuyi University, Jiangmen, Guangdong 529000, PR China.

[c]MOE Key Laboratory of Bioinorganic and Synthetic Chemistry, GBRCE for Functional Molecular Engineering, School of Chemistry, IGCME, Sun Yat-Sen University, Guangzhou 510275, PR China.

*Corresponding author. Email: wyuchemyjw@126.com; wyuchemcling@126.com.

1. Materials and Methods

General Methods. All reagents and solvents were commercially available and used without further purification. Elemental analysis (EA) was recorded by a Vario EL cube elemental analyzer. Powder X-ray diffraction (PXRD) patterns were recorded by a Rigaku Mini diffractometer. All of the simulated PXRD patterns were obtained by analyzing the CIF files of SCXRD data using the Mercury software. Room-temperature solid-state UV-vis absorption spectra were recorded on a PerkinElmer Lambda950 UV-vis-NIR absorption spectrometer coupled with an integrating sphere by using $BaSO_4$ powder as the reflectance reference. Thermogravimetric (TG) analyses were performed by a Netzsch TG209F1 Libra R instrument with a ramping rate of 5.0 °C min^{-1} under N_2 atmosphere. Gas sorption isotherms were recorded on with a Bell-Max II instrument, and the measurement temperature was maintained by liquid nitrogen (77 K), dry ice (195 K) and water bath (278 and 298 K). 0.1 M HCl and 0.1 M NaOH aqueous solutions were used to adjust the pH for the sample-immersing experiment, and the pH values was recorded using a pH meter (Mettler-Toledo SD20 Organic Kit, equipped with InLab Science Pro-ISM). Scanning electron microscopy (SEM) was performed by a Zeiss Sigma500 SEM apparatus. The wetting behavior of water on the sample surface was determined using a contact angle meter (HYK-Z-103, Hongjin Instrument Technology). The measurements were performed at 298 K, and the reported contact angles for CuIDPO represent the average values obtained from both edges of the droplet.

PXRD Patterns of CuIDPO in CO_2/N_2 Mixtures of Different Ratios (*v*/*v*). PXRD patterns of CuIDPO in CO_2/N_2 mixtures of different ratios (*v*/*v*) were collected by Ultimalv (Rigaku) powder X-ray diffractometer equipped with a rotating anode Cu K*α* X-ray generator and gas mixing control unit. The target mixture gas was blown into the sample chamber, and the sample was kept in that atmosphere for 5 minutes before collecting the PXRD patterns. After collecting the PXRD pattern, the mixture gas was evacuated using a vacuum pump, and a new target mixture gas was introduced. The PXRD patterns were then collected after the sample was kept in the new atmosphere for another 5 minutes.

Dynamic adsorption-desorption tests: Gravimetric measurements were performed using a Waters LLC TGA55 thermogravimetric analyzer under a N_2 atmosphere with or without water vapor (RH 6.4–47.3%). Platinum pans and a gas flow rate of 20 mL·min^{-1} were employed. The weight changes during water vapor adsorption and desorption processes were monitored isothermally at 298 K.

Single-Crystal X-Ray Diffraction. Diffraction data were collected on a Rigaku XtaLAB single-crystal X-ray diffractometer by using Cu-K*α* radiation (λ = 1.54184 Å). The structures were solved with the direct methods and refined with the full-matrix least-squares method on F^2 using the SHELXTL package. SHELXT was used for the structure solution of the crystals. All non-hydrogen atoms were refined with anisotropic displacement parameters, and hydrogen atoms were placed in the idealized positions and refined as rigid atoms with

the relative isotropic displacement parameters. Additional crystallographic information is shown in Tables S1 and S2. CCDC 2393520-2393525 contain the supplementary crystallographic data for this paper. These data can be obtained free of charge from the Cambridge Crystallographic Data Centre via www.ccdc.cam.ac.uk/data_request/cif.

For CuIDPO·CO_2 (291 K), in the host framework, one benzene ring exhibits disorder. The C0 C5 C4 C1 C3 C6 atoms were restrained by "ISOR 0.01". In addition, due to the less than ideal resolution and the disorder of CO_2, DFIX restraints were applied to keep the bond length (C-O bond 1.2 Å) in reasonable ranges. The occupancy of CO_2 was fixed to 0.4. The geometry and displacement parameter restraints were handled with the "DANG" and "ISOR" commands.

For CuIDPO·CO_2 (150 K), "DIFX" command was used to keep the bond length (C-O bond 1.2 Å) in reasonable ranges. The occupancy of CO_2 was fixed to 0.8. The geometry and displacement parameter restraints were handled with the "DANG" and "ISOR" commands.

Photoluminescence Measurement. The emission spectra were recorded by an Ocean QE Pro charge coupled device (CCD) equipped with a 365 nm LED. All instrument parameters such as the excitation split, emission split and scanning speed were fixed during the in-situ measurements. Variable temperature emission spectra and decay curves were obtained by the Edinburgh FLS1000 fluorescence spectrometer equipped with a continuous Xe lamp and an Oxford temperature controller. The luminescence lifetime experiments were performed by the same spectrometer equipped with a variable pulsed laser (VPL) at 375 nm as the excitation source. The experimental data were then fitted according to the following equation.[1]

$$\tau(\mathrm{obs}) = \frac{1+\frac{1}{3}\exp(-\frac{\Delta E_{\mathrm{ST}}}{k_{\mathrm{B}}T})}{\frac{1}{\tau(\mathrm{T}_1)}+\frac{1}{3\tau(\mathrm{S}_1)}\exp(-\frac{\Delta E_{\mathrm{ST}}}{k_{\mathrm{B}}T})} \quad \text{eq (S1)}$$

where τ(obs), $\tau(S_1)$, $\tau(T_1)$, k_B, T and ΔE_{ST} represent the observed lifetime, singlet state decay lifetime, triplet state decay lifetime, Boltzmann constant, temperature and singlet-triplet energy gap, respectively.

CO_2 Sensing Property Measurement. The CO_2 fluorescence sensing properties were measured in situ by placing CuIDPO inside an Oxford variable-temperature device with a four-way valve that connected the sample chamber to a pressure gage, a vacuum pump and a CO_2 cylinder. The pressure of CO_2 was recorded by Sensor PIZA 111. The emission spectra were recorded by an Ocean QE Pro charge coupled device (CCD) equipped with

a 365 nm LED. All instrument parameters such as the excitation split, emission split and scanning speed were fixed during the in-situ measurements.

Time-Dependent Photoluminescence of CH_3CN Vapor. CuIDPO was attached to a quartz slide and placed in a quartz cuvette (1.25 × 1.25 × 4.5 cm^3) (Fig. S35). The emission spectra without solvent were recorded, then 100 μL CH_3CN solution were carefully added to the cuvette, and the luminescence spectra were recorded at different times. The emission spectra were recorded by an Ocean QE Pro charge coupled device (CCD) equipped with a 365 nm LED. All instrument parameters such as the excitation split, emission split and scanning speed were fixed during the in-situ measurements.

Preparation of CuIDPO·CH_2Cl_2. CuI (0.038 g, 0.20 mmol) and DPO (bis(2-diphenylphosphinophenyl)ether, 0.108 g, 0.20 mmol) were mixed with 10 mL CH_2Cl_2 in a 25 mL glass bottle reactor. Then they were mixed adequately by 3 min ultrasonic dissolving and stood to get clear solution. The transparent crystals were collected after 24 h. At last, they were washed with CH_2Cl_2 for three times (yield 60%). Elemental analysis calcd (%) for $C_{72}H_{56}Cu_2I_2O_2P_4$: C 59.31, H 3.87; found: C 59.68, H 4.19.

Preparation of CuIDPO. The CuIDPO·CH_2Cl_2 was dried under vacuum at room temperature for 4 h to get the guest-free CuIDPO single crystals.

Preparation of CuIDPO@GF. Firstly, CuI (0.019 g, 0.10 mmol) was dissolved in CH_3CN (5 mL), and then sprayed on the glass fiber (GF) paper, and dried in the air. Secondly, dry GF paper with CuI were immersed in a CH_2Cl_2 solution of DPO (0.054 g, 0.10 mmol). The DPO was reacted with CuI to form CuIDPO·CH_2Cl_2 powder inside the GF paper. At last, after washing the GF paper 3 times with CH_2Cl_2, a vacuum pump was used to remove the CH_2Cl_2 from the paper.

Density Functional Theory (DFT) Calculations. DFT calculations were performed using Gaussian 09 program.[2] Geometry optimizations were performed using the PBE0 density functional with D3 dispersion correction.[3] The 6-31G(d) basis set was used for C, O, P and H, while LanL2DZ pseudopotential was used for Cu and I. The unrestricted DFT calculations were employed to optimize the T_1 structure with the same function and basis set.

Time-Dependent Density Functional Theory (TDDFT) Calculations. TDDFT calculations were used to investigate the photophysical properties of the compounds. The calculations were performed with the same function and basis set as described for the DFT optimizations. The first 15 singlet states without geometry optimization were calculated for the absorption transition based on the S_0 structure. The first 5 singlet states with geometry optimization were calculated for the fluorescence transition based on the S_0 structure. The first 5 triplet states without geometry optimization were calculated for the phosphorescence transition of $T_1 \rightarrow S_0$ based on the T_1 structure, which was based on the unrestricted DFT calculation. The Milliken orbital composition analyses were calculated by the Multiwfn

program.[4] The VMD (Visual Molecular Dynamics) software is used for reading, visualizing, and creating data images obtained from Gaussian calculations.[5] The atomic coordination are shown in Table S5-6.

Calculations of Binding Energies between CO_2/CH_3CN and Adjacent Phenyl Rings. Periodic DFT was calculated by Dmol3 module of Materials Studio 8.0 software package. Geometry optimizations were used to get the precise structure, original from crystal structure. And the non-hydrogen atoms of the host framework were fixed during the geometry optimizations. The Perdew-Burke-Ernzerhof (PBE) generalized gradient approximation (GGA) exchange–correlation functional with the double numeric plus (DNP) polarization basis set was utilized.[6] The effective core potentials were used. Grimme semiempirical methods to describe the long-range van der Waals interactions. The DFT-D are employed to the dispersion correction.[7] The convergence tolerance was as follows: 10^{-5} Ha (Energy), 0.002 Ha/Å (Max. force) and 0.005 Å (Max. displacement).

Then the corresponding structural segments (one hydrogen atom was added to change phenyl group into benzene for convenience) were performed by Gaussian 09 program, based on the above period structure. Binding energies were performed using by PBE0 density function with D3 dispersion correction.[3] The 6-31G(d) basis set was used for C, O and H The binding energy (ΔE) was computed by the following equation: $\Delta E = E$ (benzene and guest) $- E$ (benzene) $- E$ (guest). The calculation results are shown in Table S4. The atomic coordination are shown in Table S7-8.

2. Figures and Tables

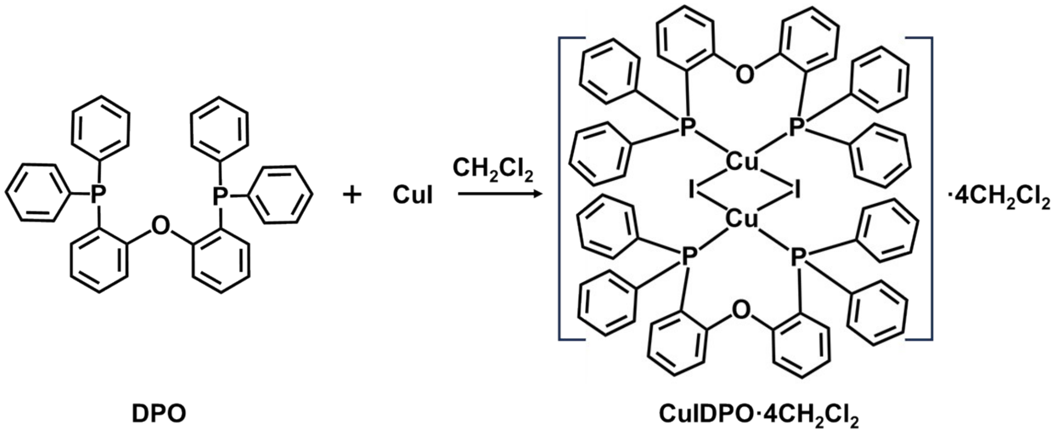

Fig. S1. Schematic diagram of the synthesis of $CuIDPO \cdot CH_2Cl_2$.

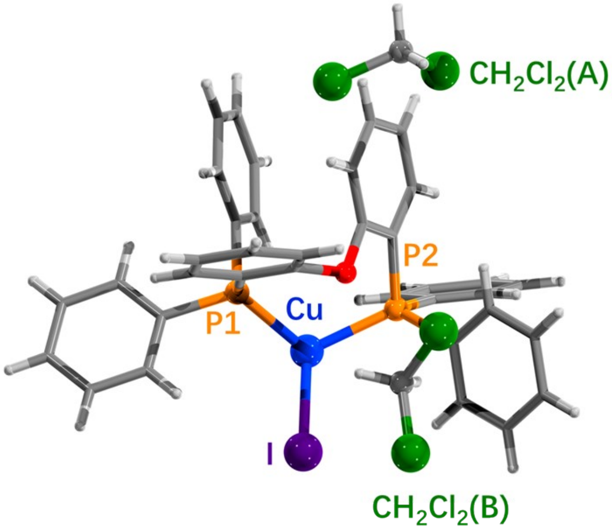

Fig. S2. An asymmetric unit of $CuIDPO \cdot CH_2Cl_2$ (SCXRD data at 150 K). Color codes: Cu, blue; I, purple; P, orange; C, grey; O, red; H white; Cl, green.

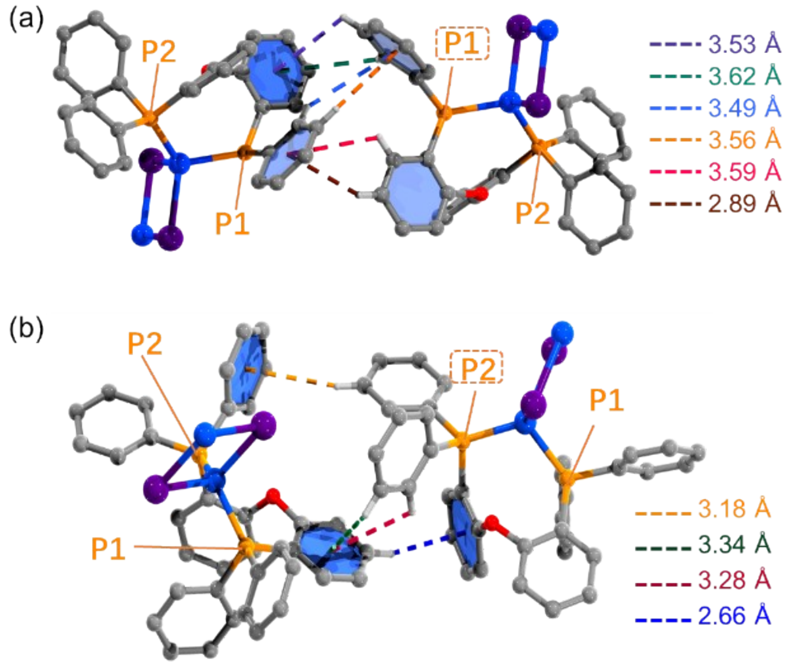

Fig. S3. The C–H···π interactions of phenyl rings linked to (a) P1 and (b) P2 of an adjacent CuIDPO (SCXRD data at 291.6K) cluster. Color codes: Cu, blue; I, purple; P, orange; C, grey; H white; O, red. Hydrogen atoms without interaction sites are omitted for clarity.

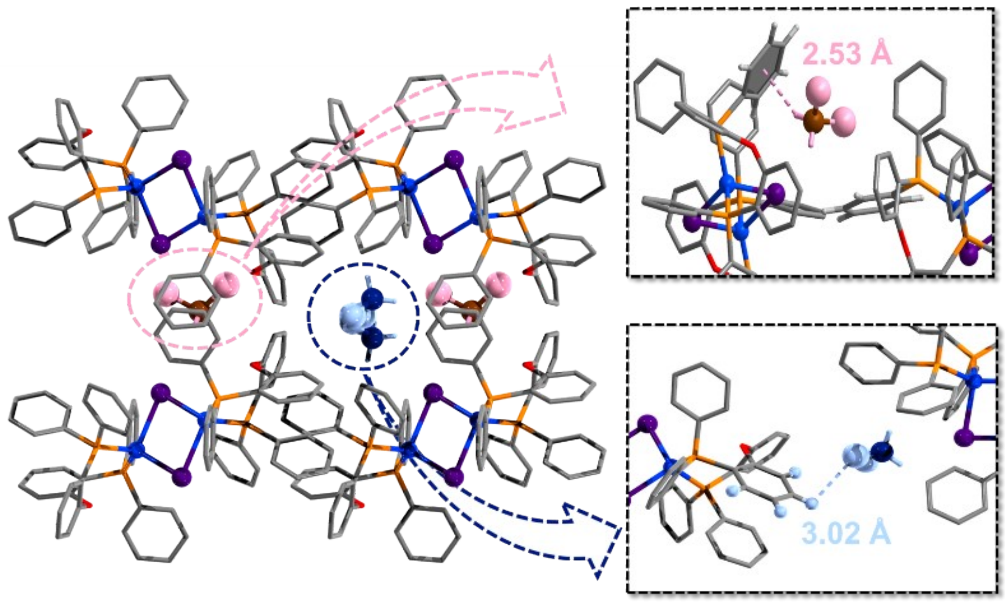

Figure S4. Molecular stacking model in CuIDPO·CH_2Cl_2 (SCXRD data at 150 K) viewed along the *c*-axis. The distance of C–H···π between CH_2Cl_2(A) (pink) and its adjacent phenyl ring is 2.53 Å, while C–H···Cl between the CH_2Cl_2(B) (blue) and its adjacent phenyl ring is 3.02 Å. Color codes: Cu, blue; I, purple; P, orange; C, grey; O, red. Hydrogen atoms without interaction sites are omitted for clarity.

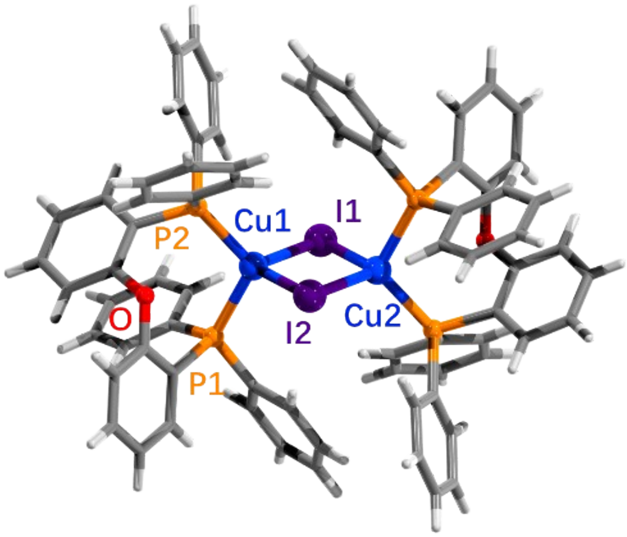

Fig. S5. Molecular structure of CuIDPO (SCXRD data at 291.6 K). Color codes: Cu, blue; I, purple; P, orange; C, grey; H white; O, red.

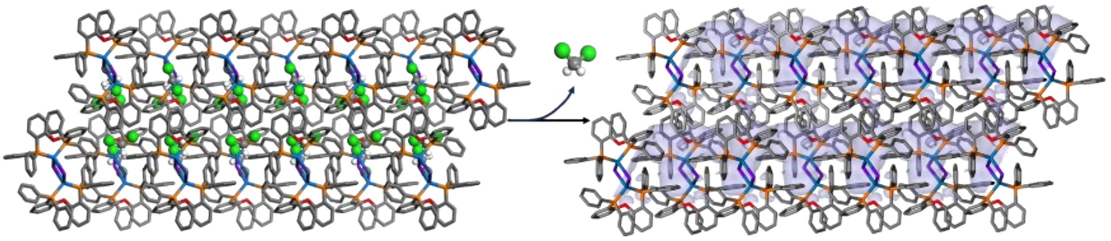

Fig. S6. Comparison of the framework and pore surface structure of CuIDPO·CH_2Cl_2 (SCXRD data at 150 K) (left) and guest-free CuIDPO (SCXRD data at 150 K) (right) after removing guest CH_2Cl_2. Except the guest molecules, hydrogen atoms are omitted for clarity.

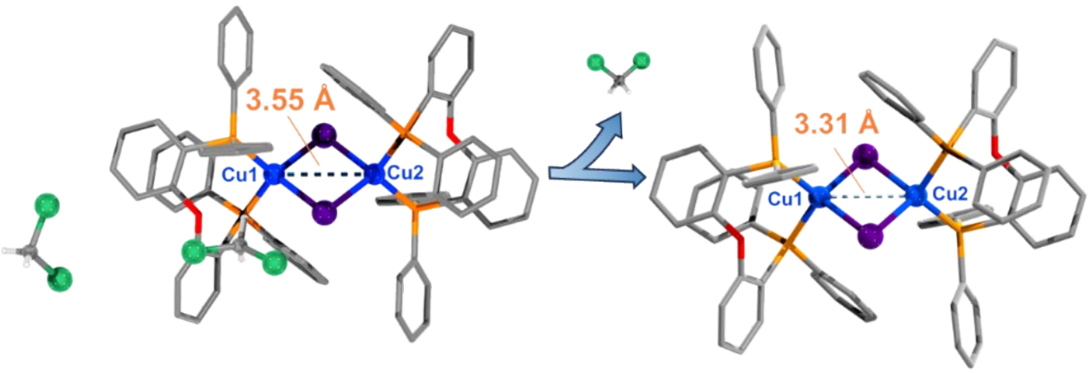

Fig. S7. Comparison of the intramolecular Cu–Cu distance in CuIDPO·CH_2Cl_2 (SCXRD data at 150 K) (left) and guest-free CuIDPO (SCXRD data at 150 K) (right). Color codes: Cu, blue; I, purple; P, orange; C, grey; H white; O, red; Cl, green. Except the guest molecules, hydrogen atoms are omitted for clarity.

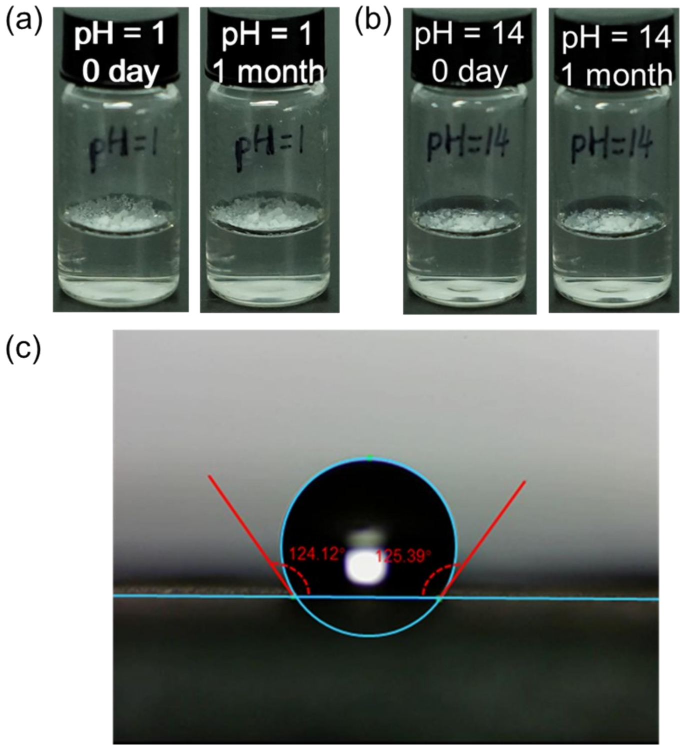

Fig. S8. Photographs of CuIDPO before and after immersion in the aqueous solutions of (a) pH = 1 and (b) pH = 14 for 1 month. (c) Photograph of CuIDPO in the contacting angle test. The average of contact angles is 124.75°.

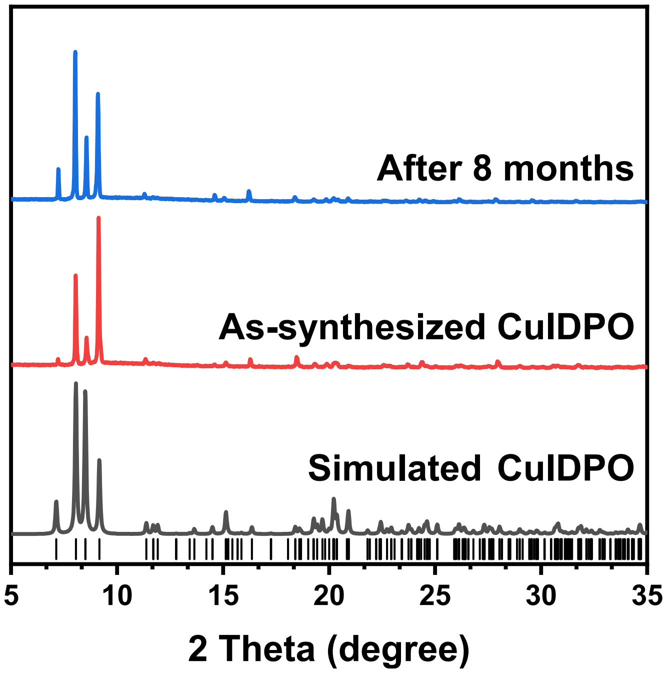

Fig. S9. PXRD patterns of as-synthesized CuIDPO and that after exposure in air for 8 months.

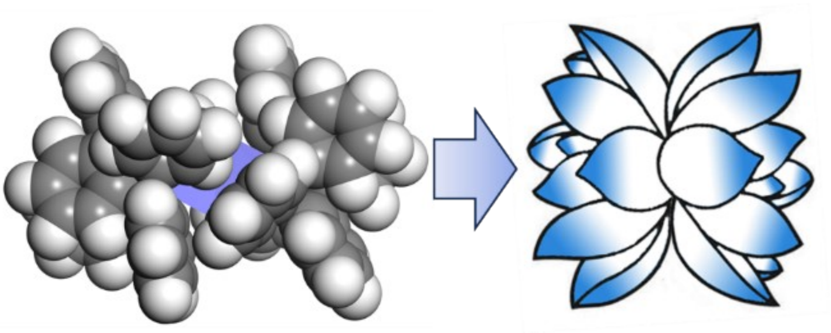

Fig. S10. The petal-like structure of CuIDPO (SCXRD data at 291.6 K). Color codes: Cu, blue; I, purple; P, orange; C, grey; H white; O, red.

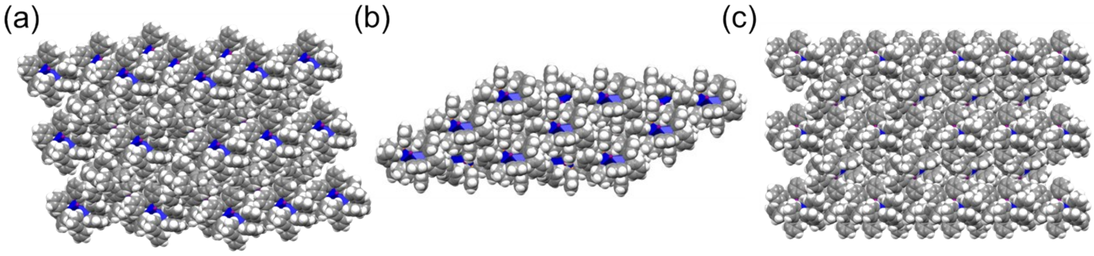

Fig. S11. (a-c) The (100), (010) and (001) crystal planes of CuIDPO (SCXRD data at 291.6 K). Color codes: Cu, blue; I, purple; P, orange; C, grey; H white; O, red.

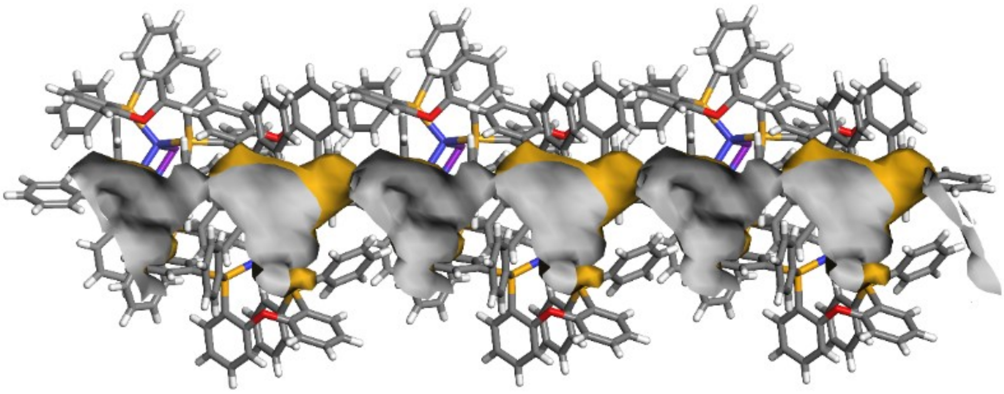

Fig. S12. Structure of the hydrophobic pore surface of guest-free CuIDPO (SCXRD data at 291.6 K). Color codes: Cu, blue; I, purple; P, orange; C, grey; H white; O, red.

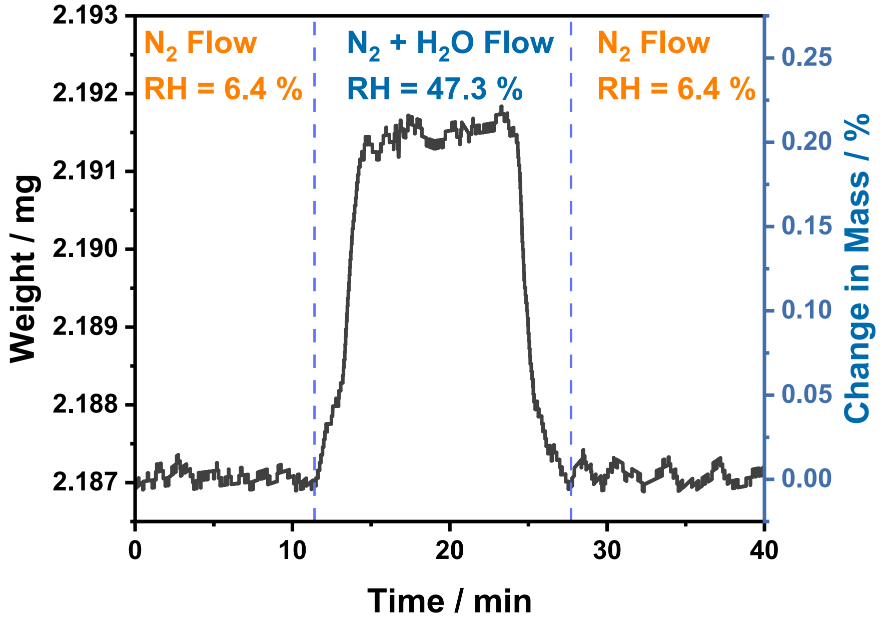

Fig. S13. Dynamic adsorption−desorption TGA curve of CuIDPO.

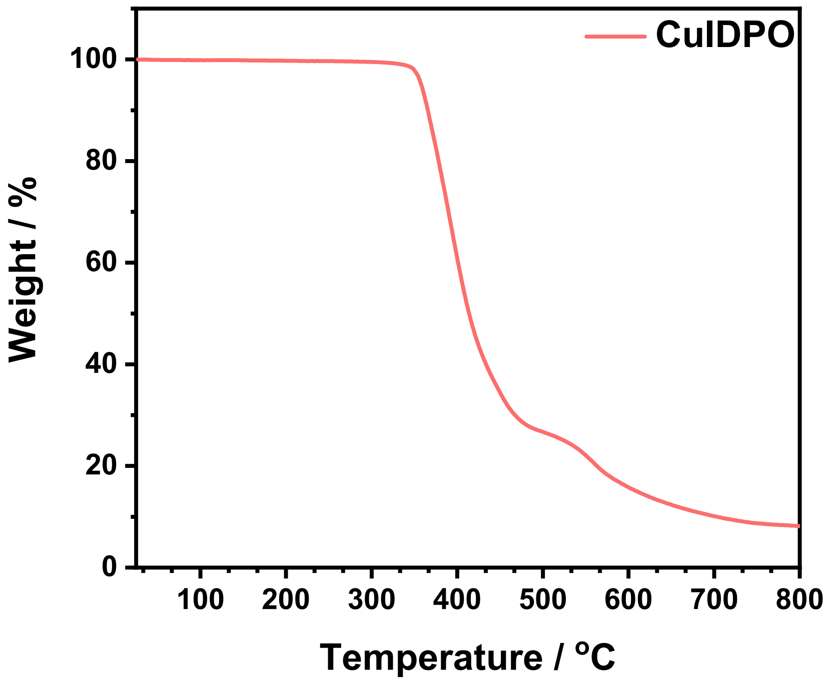

Fig. S14. Thermogravimetric (TG) curve of CuIDPO.

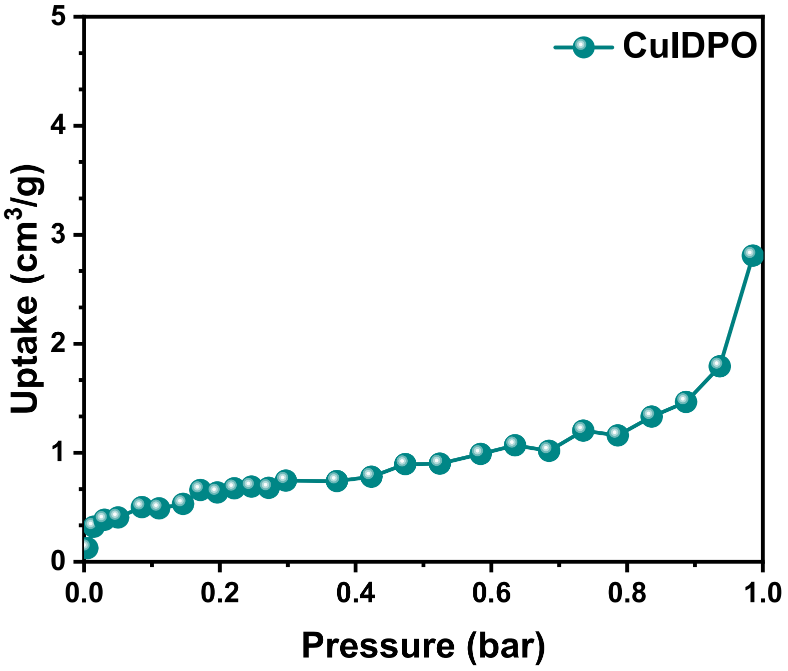

Fig. S15. N_2 sorption isotherm for CuIDPO at 77 K.

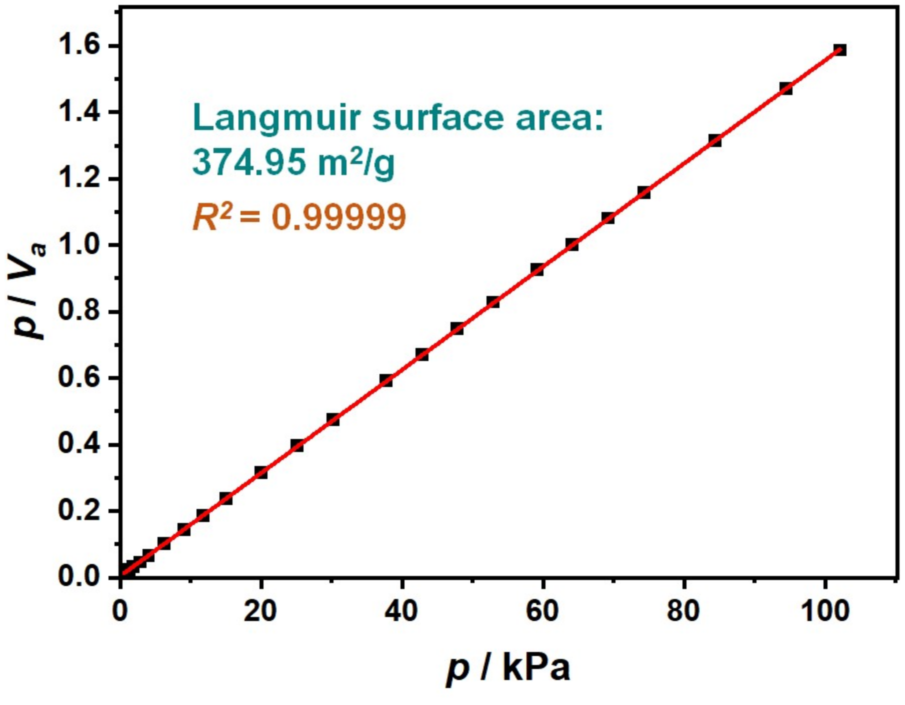

Fig. S16. The fitting curve of Langmuir surface area based on CO_2 sorption isotherm at 195 K.

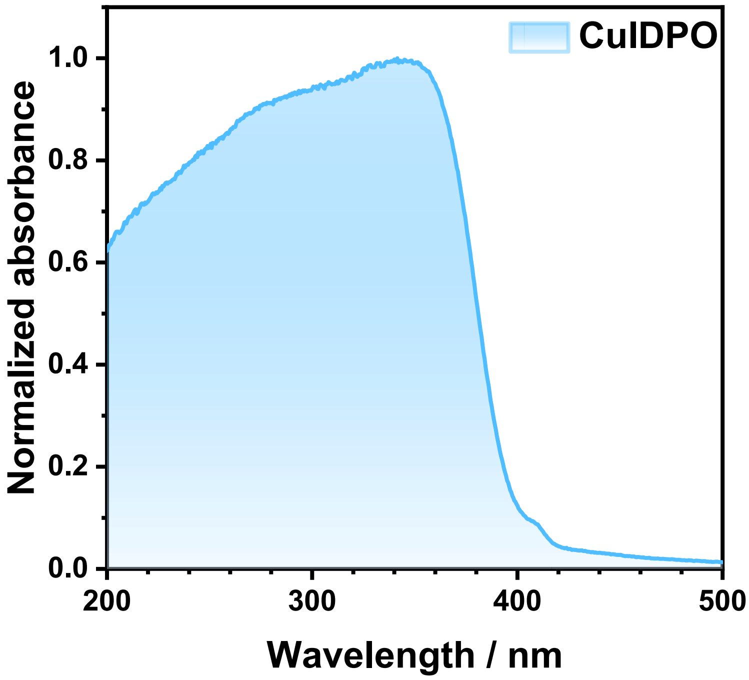

Fig. S17. UV-vis adsorption spectra of CuIDPO at room temperature.

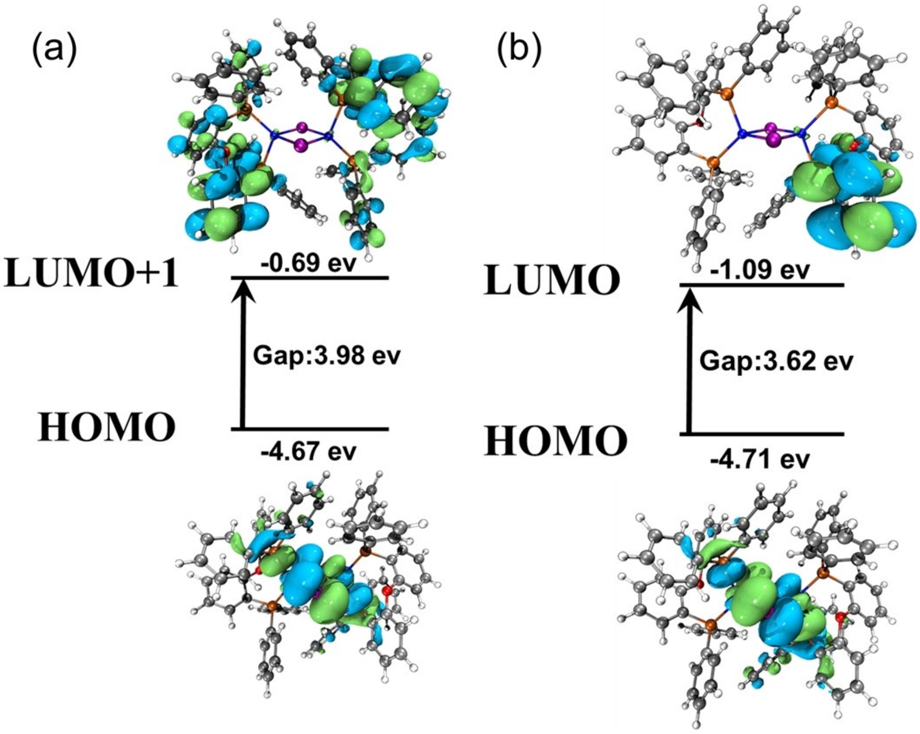

Fig. S18. (a) The $S_0 \rightarrow S_2$ and (b) $S_0 \rightarrow T_1$ transitions of CuIDPO, evaluated by DFT and TDDFT calculations.

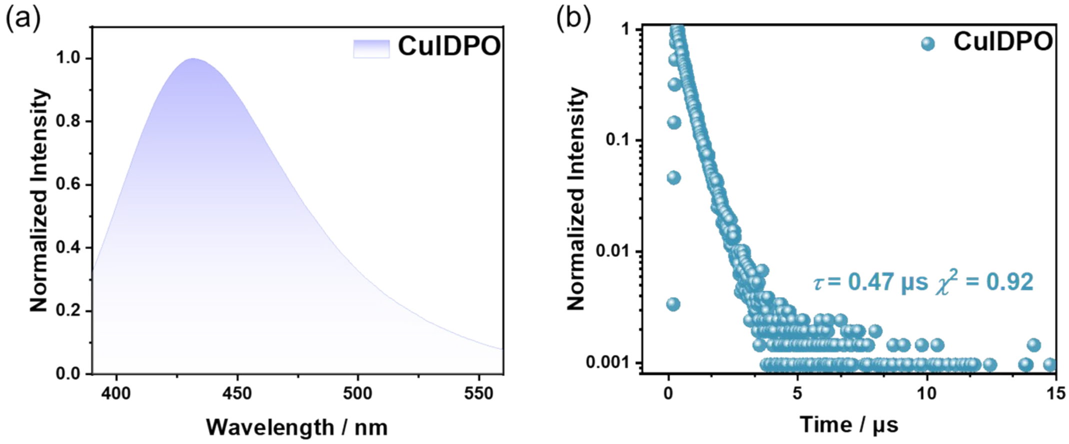

Fig. S19. (a) Emission spectra of CuIDPO in air, excited at 365 nm. (b) Luminescence decay curves of CuIDPO in air, excited by 375 nm VPL and detected at 432 nm.

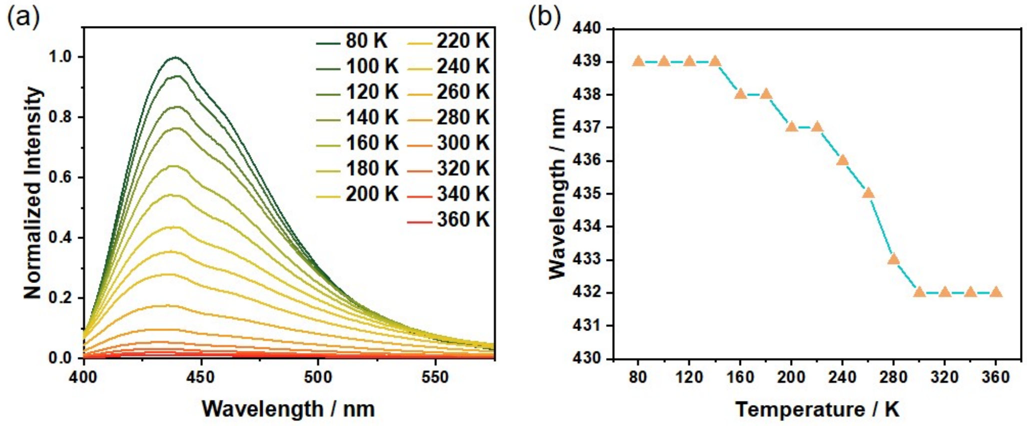

Fig. S20. (a) Emission spectra of CuIDPO at different temperatures between 80 K and 360 K, excited at 365 nm. (b) The plot of emission peak maximum (λ_{em}) against temperature based on (a).

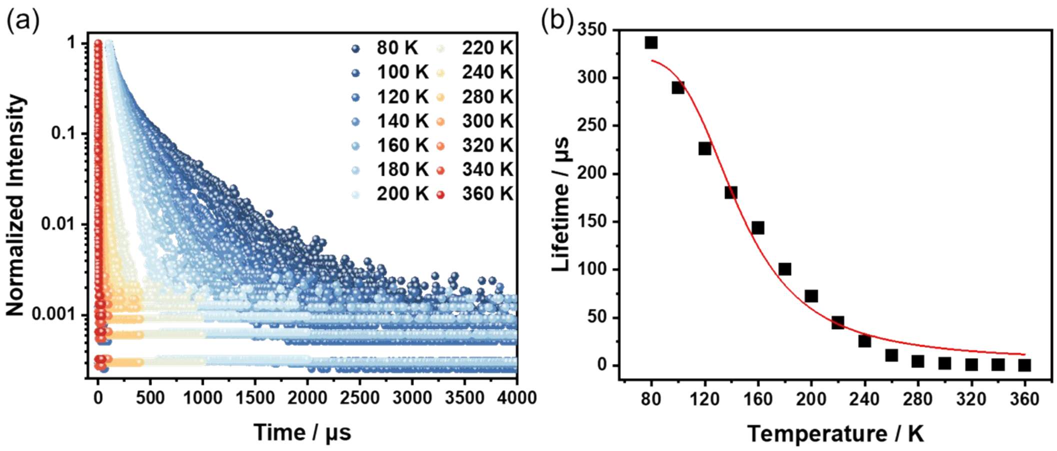

Fig. S21. (a) Luminescence decay curves of CuIDPO between 80 K and 360 K, excited by 375-nm VPL and detected at 432 nm. (b) Plot of emission decay time against temperature; red line represents the fit according to eq (S1).

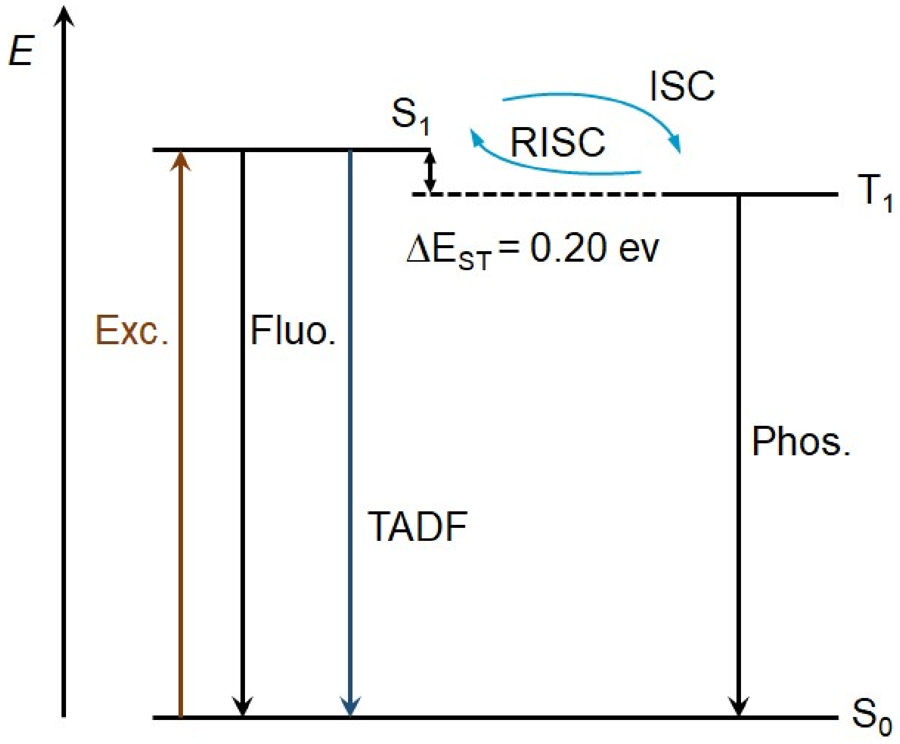

Fig. S22. Simplified Jablonski diagram of CuIDPO TADF emitter based on the DFT and TDDFT calculations. S_1, T_1, S_0, ISC and RISC represent the first singlet excited state, the first triplet excited state, ground state, intersystem conversion, reverse intersystem conversion, respectively.

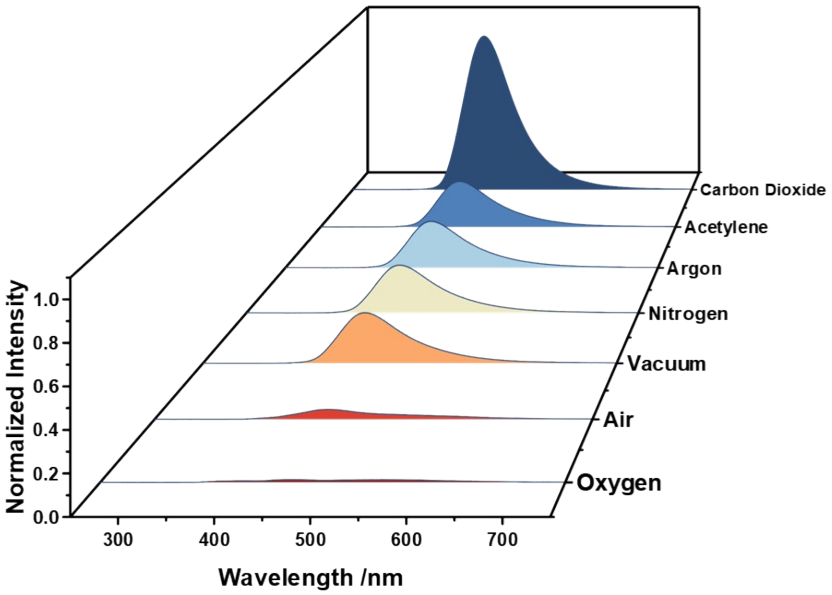

Fig. S23. Emission spectra of CuIDPO in different gases, excited by 365 nm LED.

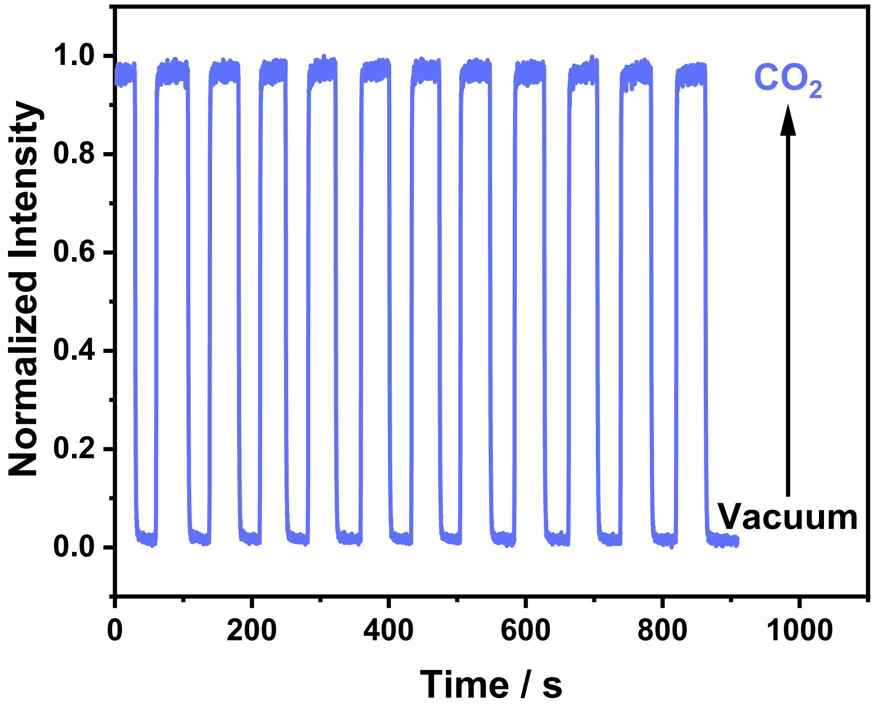

Fig. S24. Kinetic scan of CuIDPO when alternating vacuum and CO_2, excited at 365 nm and detected at 432 nm.

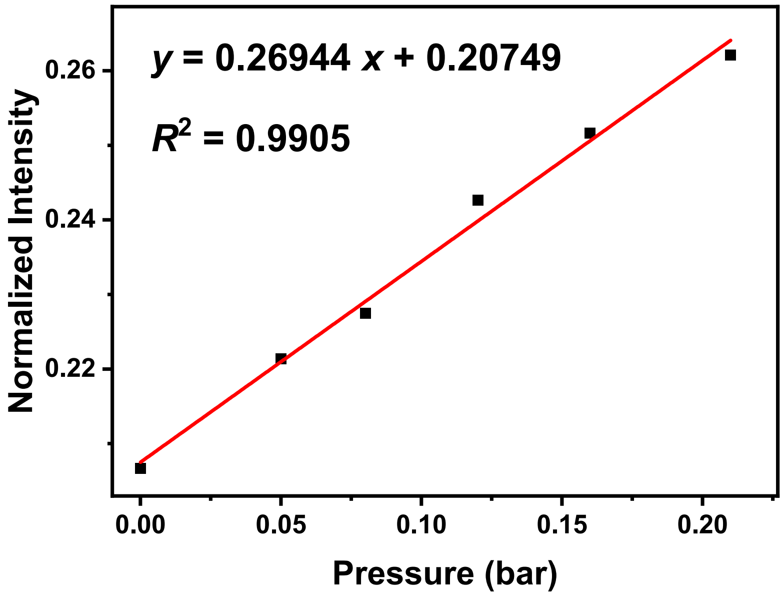

Fig. S25. The luminescence intensity of CuIDPO in 0.034 mbar to 210 mbar CO_2. The curve was fitted according to the linear function ($y = k \times x + b$). The LOD were defined at 1% enhancing to ensure comparability, which makes them not subject to specific instrumental characteristics or artificial operation error. We define the point that CO_2 begins to enhance the luminescence as (x_0, y_0) in the linear function. We think that the intensity of the x value of LOD (x_{LOD}) will increase to $1.01y_0$, meaning the luminescence has been enhanced 1%. Therefore, $1.01y_0 = k \times x_{LOD} + b$, $y_0 = k \times x_0 + b$, $x_{LOD} = x_0 + y_0 / 100k$ eq(S2).

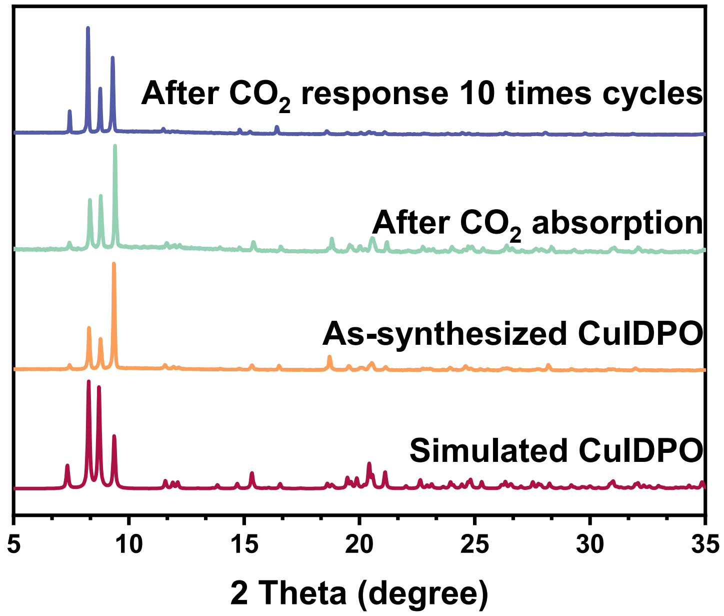

Fig. S26. PXRD patterns of as-synthesized CuIDPO and those after the CO_2 adsorption experiments and CO_2 sensing experiments.

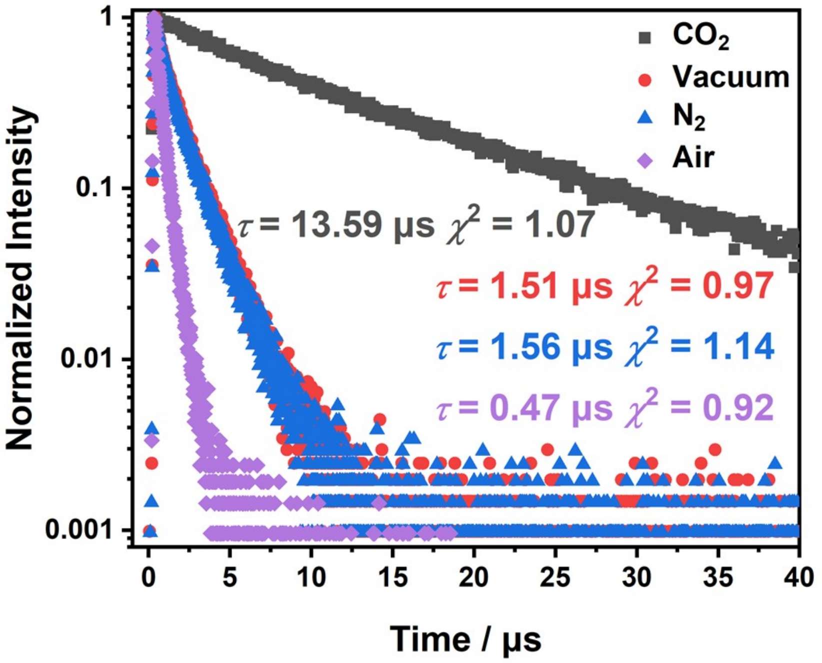

Fig. S27. The decay curves of CuIDPO in different gas atmospheres at room temperature, excited by 375-nm VPL and detected at 432 nm.

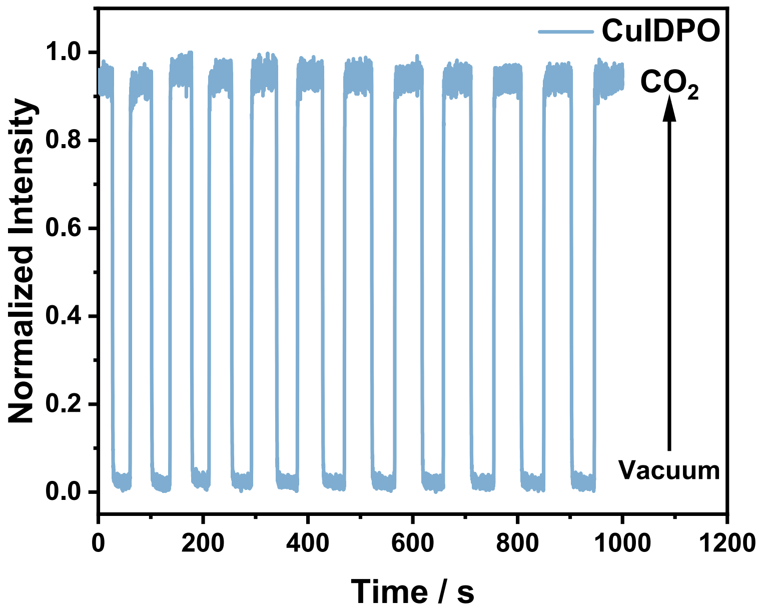

Fig. S28. Kinetic scan of CuIDPO after immersion in the aqueous solutions at pH = 1 for 1 week when alternating vacuum and CO_2, excited at 365 nm and detected at 432 nm.

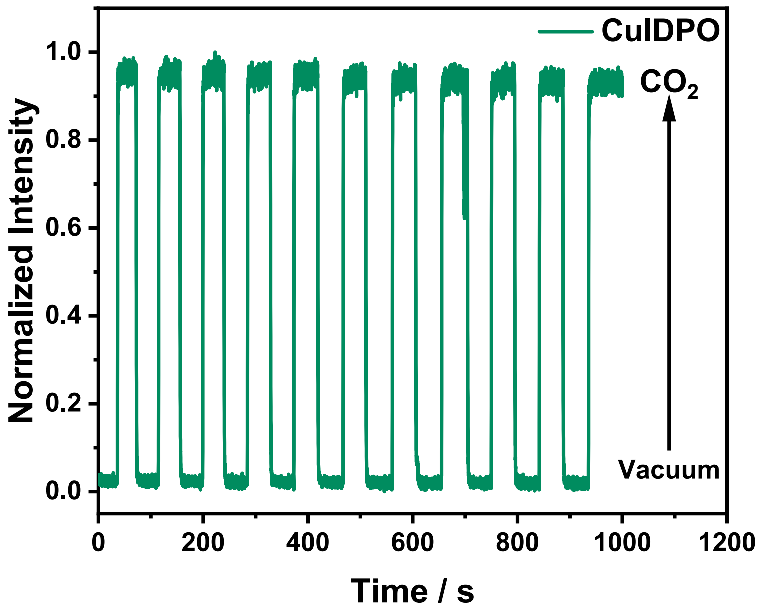

Fig. 29. Kinetic scan of CuIDPO after immersion in the aqueous solutions of pH = 14 for 1 week when alternating vacuum and CO_2, excited at 365 nm and detected at 432 nm.

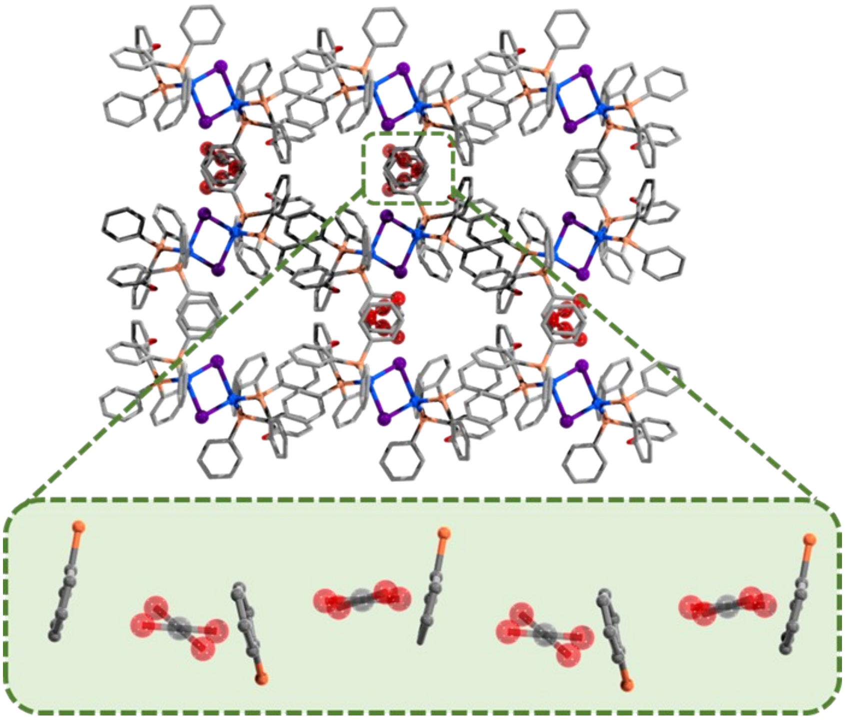

Fig. S30. Crystal structure of CuIDPO·CO_2 at 291 K. Color codes: Cu, blue; I, purple; P, orange; C, grey; O, red. All hydrogen atoms are omitted for clarity.

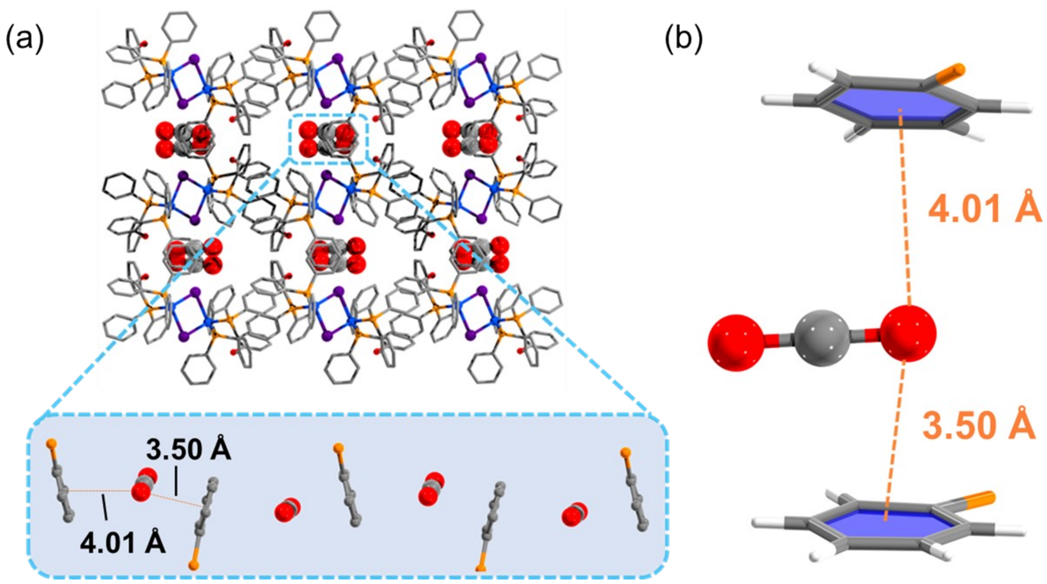

Fig. S31. (a) Crystal structure of CuIDPO·CO_2 at 150 K. (b) The π–π interaction between CO_2 and phenyl ring in CuIDPO·CO_2. Color codes: Cu, blue; I, purple; P, orange; C, grey; H white; O, red. Some hydrogen atoms are omitted for clarity.

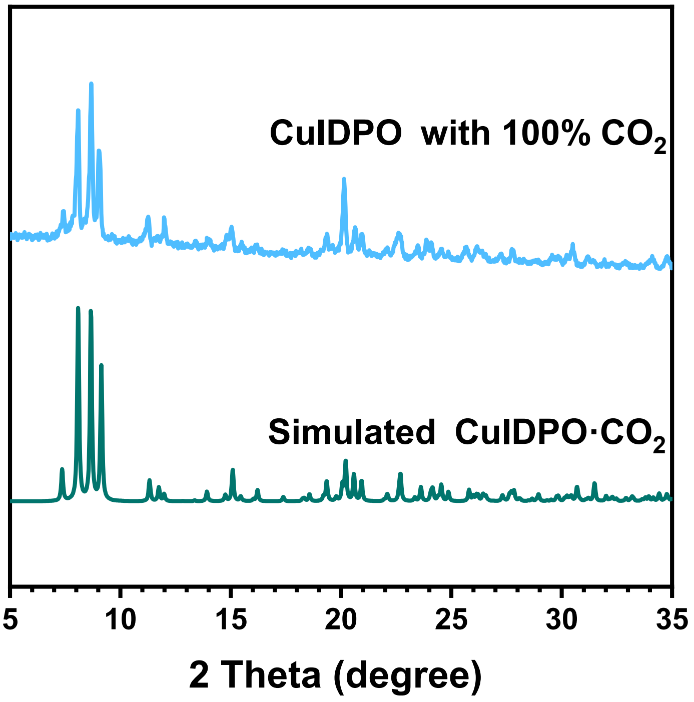

Fig. S32. PXRD patterns of CuIDPO in 100% CO_2 at room temperature.

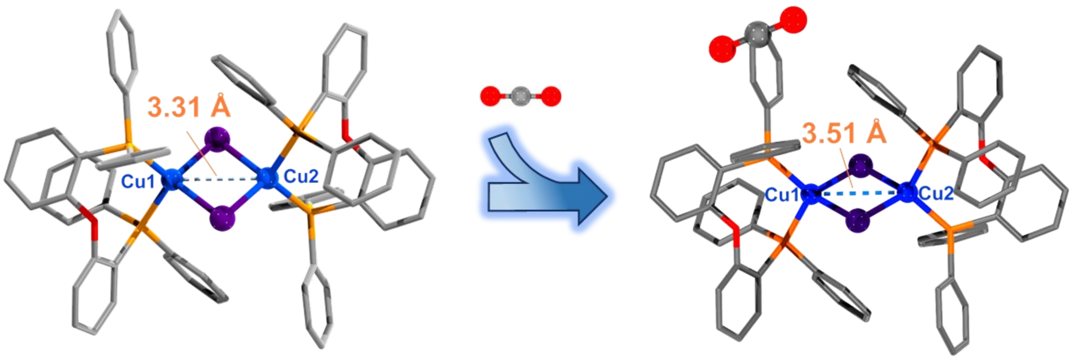

Fig. S33. The Cu···Cu distances of guest-free CuIDPO (SCXRD data at 150 K) and CuIDPO·CO_2 (SCXRD data at 150 K). Color codes: Cu, blue; I, purple; P, orange; C, grey; O, red. All hydrogen atoms are omitted for clarity.

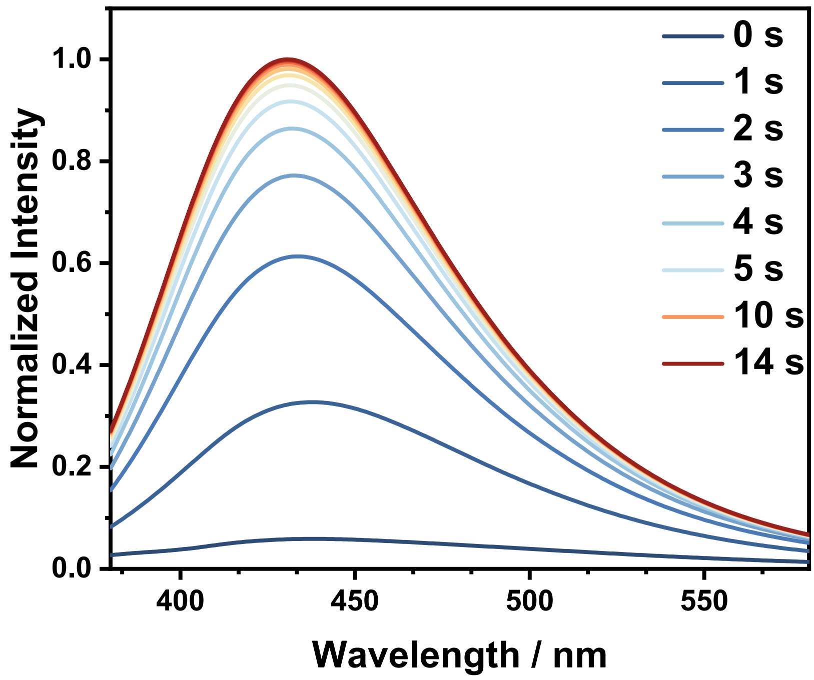

Fig. S34. Time-dependent emission spectra of CuIDPO after exposure in CH_3CN saturated vapor, excited by a 365 nm LED.

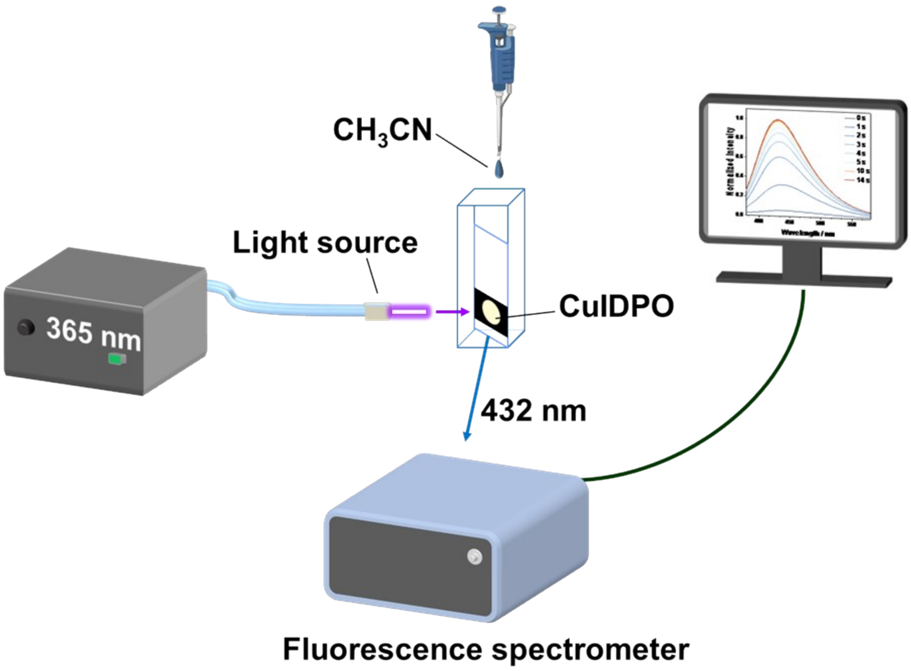

Fig. S35. Schematic diagram of the in-situ solid state luminescence sensing for CH_3CN.

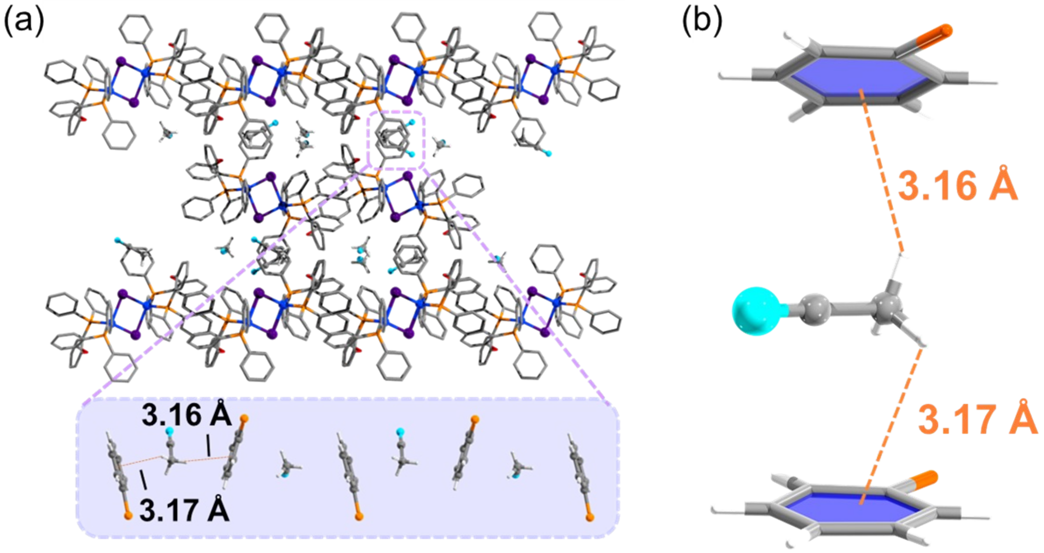

Fig. S36. (a) The crystal structure of CuIDPO·CH_3CN at 150 K. (b) The C–H···π interaction between CH_3CN and phenyl ring of CuIDPO·CH_3CN. Color codes: Cu, blue; I, purple; P, Orange; C, grey; N, turquoise; H white; O, red. Some hydrogen atoms are omitted for clarity.

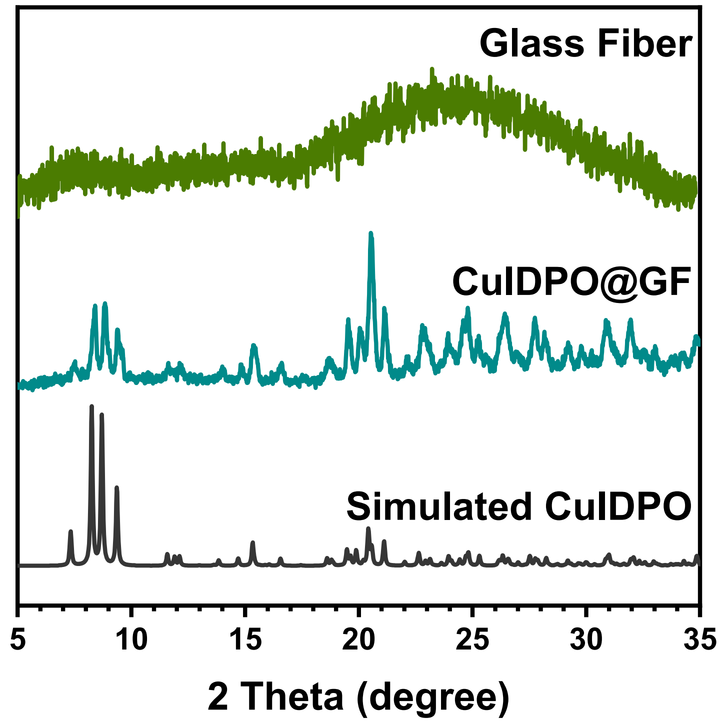

Fig. S37. PXRD patterns of CuIDPO@GF and the GF paper.

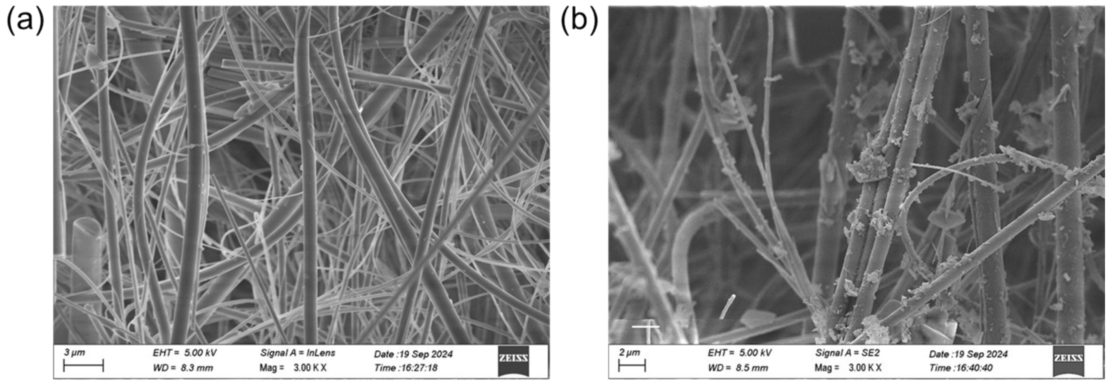

Fig. S38. SEM images of (a) GF paper and (b) CuIDPO@GF.

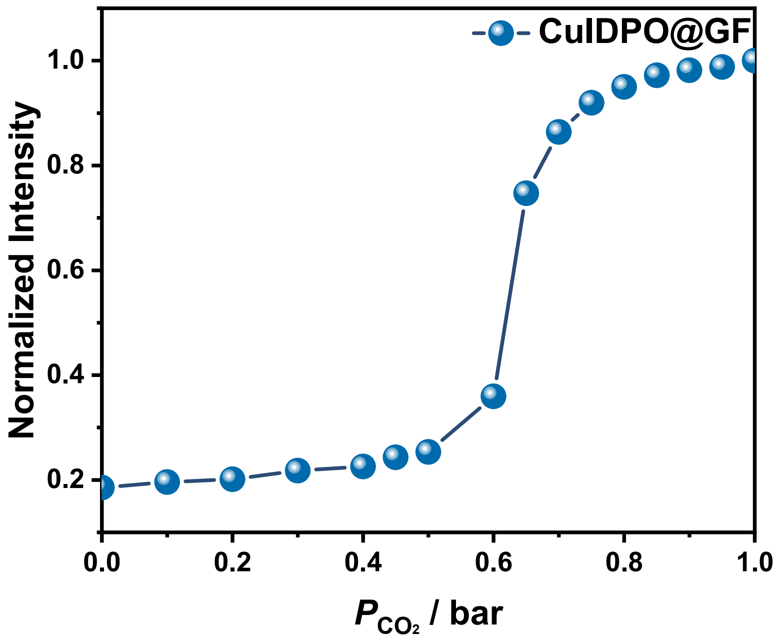

Fig. S39. The intensity enhancement curve for CuIDPO@GF in different pressures of CO_2, excited at 365 nm and detected at 432 nm.

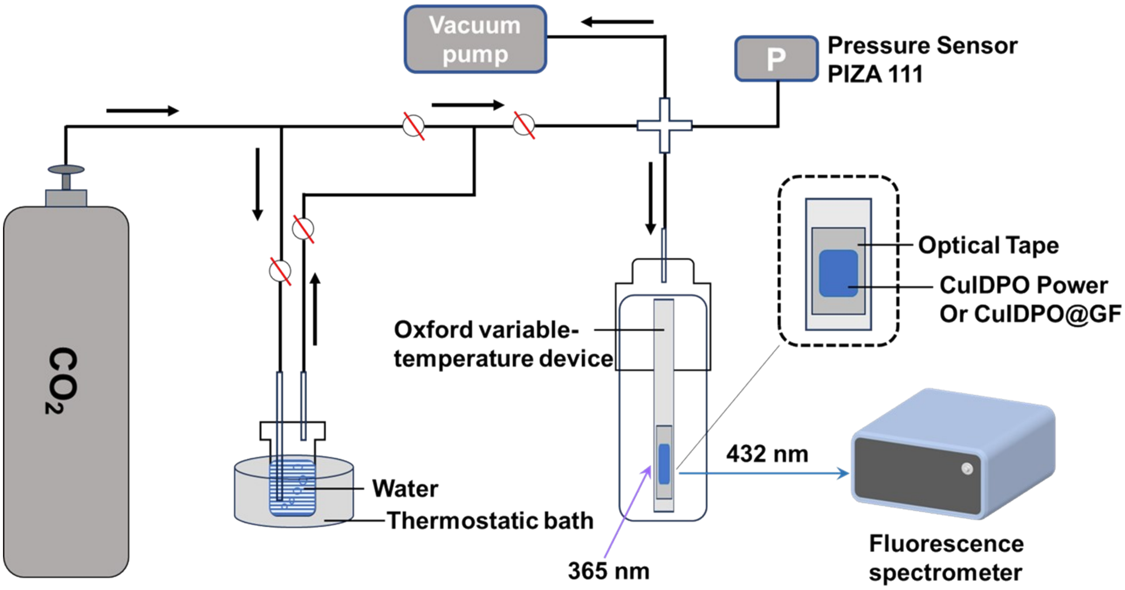

Fig. S40. Schematic diagram of the device for testing high humidity CO_2 fluorescence response.

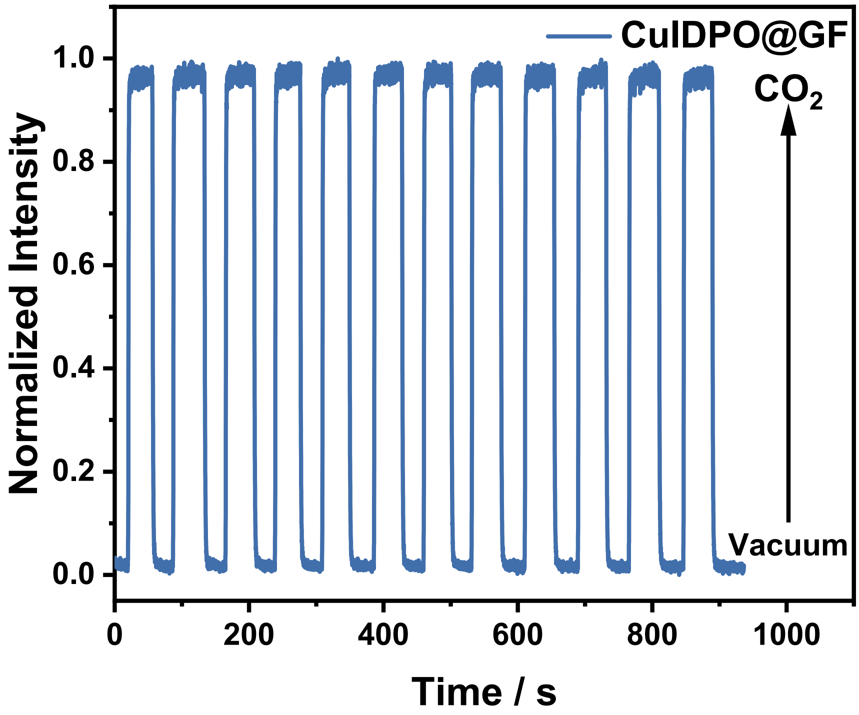

Fig. S41. Kinetic scan of CuIDPO@GF when alternating vacuum and humid CO_2, excited at 365 nm and detected at 432 nm.

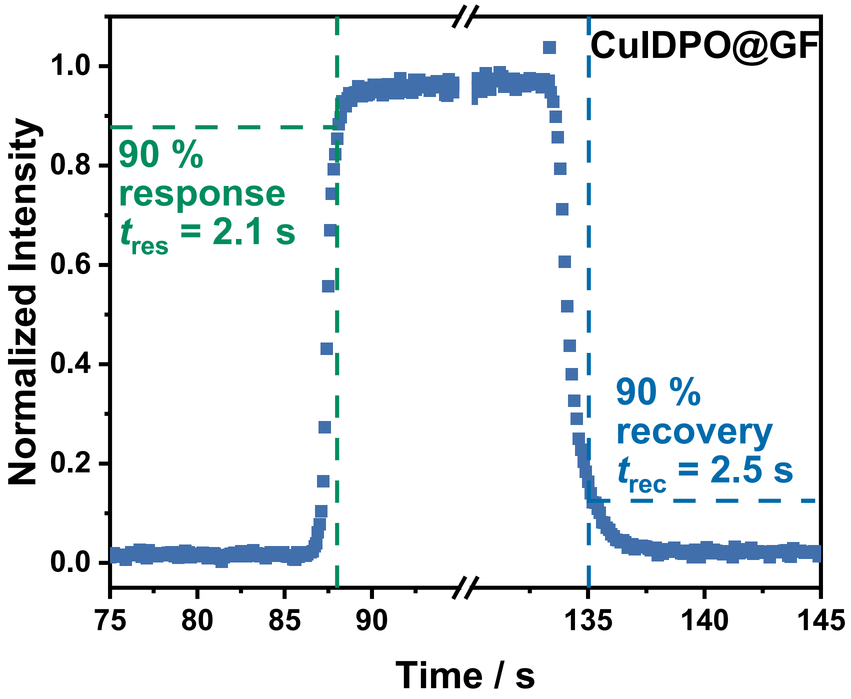

Fig. S42. Enlargement of one cycle of Fig. S41.

Table S1. Crystallographic data and structural refinement parameters.

Compounds	CuIDPO·CH_2Cl_2	CuIDPO	CuIDPO
Formula	$C_{76}H_{64}Cl_8Cu_2I_2O_2P_4$	$C_{72}H_{56}Cu_2I_2O_2P_4$	$C_{72}H_{56}Cu_2I_2O_2P_4$
Formula weight	1798	1458	1458
T / K	150.00(10)	291.6(3)	149.99(10)
Crystal system	monoclinic	monoclinic	monoclinic
Space group	$P2_1/c$	$P2_1/c$	$P2_1/c$
a / Å	13.5563(2)	13.5886(1)	13.4634(8)
b / Å	20.7370(2)	18.8697(2)	18.7343(17)
c / Å	14.8064(2)	14.6510(2)	23.9159(15)
α / °	90	90	90
β / °	116.901(2)	117.565(1)	147.569(16)
γ / °	90	90	90
Volume / Å^3	3711.92(10)	3330.27(7)	3235.0(8)
Z	2	2	2
R_{int}	0.0566	0.0470	0.0496
R_1 [$I > 2\sigma(I)$] [a]	0.0337	0.0243	0.0268
wR_2 [$I > 2\sigma(I)$] [b]	0.0881	0.0597	0.0694
R_1 (all data)	0.0337	0.0243	0.0268
wR_2 (all data)	0.0887	0.0610	0.0706
GOF	1.061	1.038	1.050

Table S2. Crystallographic data and structural refinement parameters.

Compounds	CuIDPO·CO_2	CuIDPO·CO_2	CuIDPO·CH_3CN
Formula	$C_{73.6}H_{56}Cu_2I_2O_{5.12}P_4$	$C_{72.8}H_{56}Cu_2I_2O_{3.6}P_4$	$C_{80}H_{68}Cu_2I_2N_4O_2P_4$
Formula weight	1527	1493	1622
T / K	150.00(11)	291.33(10)	150.00(11)
Crystal system	monoclinic	monoclinic	monoclinic
Space group	$P2_1/n$	$P2_1/c$	$P2_1/n$
a / Å	13.3073(1)	13.4567(1)	13.3463(5)
b / Å	19.2892(1)	19.3380(1)	20.2060(4)
c / Å	14.7456(2)	14.8468(2)	14.7519(5)
α / °	90	90	90
β / °	116.688(1)	116.934(1)	116.536(4)
γ / °	90	90	90
Volume / $Å^3$	3381.77(6)	3444.44(6)	3559.1(2)
Z	2	2	2
R_{int}	0.0343	0.0377	0.0486
R_1 [$I > 2\sigma(I)$] [a]	0.0277	0.0269	0.0355
wR_2 [$I > 2\sigma(I)$] [b]	0.0673	0.0726	0.0948
R_1 (all data)	0.0277	0.0269	0.0355
wR_2 (all data)	0.0679	0.0738	0.0967
GOF	1.149	1.066	1.050

[a] $R_1 = \Sigma||F_o| - |F_c|| / \Sigma |F_o|$

[b] $wR_2 = [\Sigma w(F_o^2 - F_c^2)^2/\Sigma w(F_o^2)^2]^{1/2}$

Table S3. The main bond lengths (Å) of crystals.

Compounds	CuIDPO· CH_2Cl_2 (150 K)	CuIDPO (291 K)	CuIDPO (150 K)	CuIDPO· CO_2 (150 K)	CuIDPO· CO_2 (291 K)	CuIDPO· CH_3CN (150 K)
Cu–Cu / Å	3.552	3.381	3.313	3.515	3.534	3.648
Cu–I / Å	2.642	2.687	2.683	2.660	2.673	2.651
Cu–P1 / Å	2.294	2.304	2.296	2.283	2.292	2.288
Cu–P2 / Å	2.283	2.290	2.279	2.268	2.278	2.275

Table S4. The binding energy of π–π and C–H···π interaction, evaluated by DFT calculations.

	DFT-D3 (Hartree)
CO_2 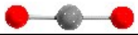	–188.5583
Benzene 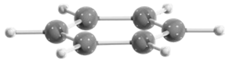	–232.2650
CO_2+ Benzene 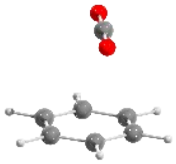	–420.8286
$\Delta E = E_{Guest+Benzene}$ - $(E_{Guest} + E_{Benzene})$	–0.0053
CH_3CN 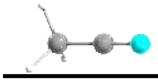	–132.7534
Benzene 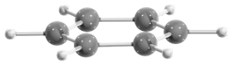	–232.2656
CH_3CN+ Benzene 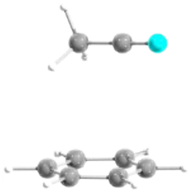	–365.0251
$\Delta E = E_{Guest+Benzene} - (E_{Guest} + E_{Benzene})$	–0.0061

Table S5. The atomic coordinates of the ground state of CuIDPO in the computational results.

Tag	Element	*x*	*y*	*z*
1	I	-0.1069170	-0.0569030	-2.0813620
2	Cu	1.6287220	-0.0444560	-0.0633350
3	P	2.4417390	2.1661670	-0.0582750
4	P	3.2995340	-1.6858520	-0.0052500
5	O	4.2856980	0.4628670	1.6040860
6	C	3.6836150	2.4688570	-1.3714990
7	C	1.2317550	3.5258650	-0.2598980
8	C	4.5522340	3.5693660	-1.3319610
9	H	4.4986630	4.2685320	-0.5015130
10	C	3.3284970	2.6710000	1.4761750
11	C	5.1905630	0.2534170	0.5806950
12	C	4.9123980	-0.8487080	-0.2421580
13	C	4.1438430	1.7343880	2.1314400
14	C	3.4880290	-2.5783130	1.5775560
15	C	3.1974910	3.9391370	2.0608960
16	H	2.5681430	4.6821670	1.5813750
17	C	3.7668730	1.5631570	-2.4353070
18	H	3.0987890	0.7059780	-2.4586340
19	C	5.4871760	3.7605890	-2.3462770
20	H	6.1576110	4.6148710	-2.3096340
21	C	0.2581970	3.7092500	0.7362340
22	H	0.2101300	3.0270680	1.5806460
23	C	5.8069230	-1.1438230	-1.2762970
24	H	5.6076180	-1.9923480	-1.9236150
25	C	3.8413790	4.2489000	3.2592860
26	H	3.7211820	5.2371440	3.6931220
27	C	3.3659530	-3.0039250	-1.2732520
28	C	4.7768330	2.0248250	3.3357950
29	H	5.3744520	1.2527580	3.8099750
30	C	7.1696050	0.7585550	-0.6799090
31	H	8.0319540	1.3942660	-0.8575860
32	C	4.7376700	-2.8059040	2.1706930
33	H	5.6399030	-2.4382480	1.6886490
34	C	5.5652500	2.8526620	-3.4060730
35	H	6.2990510	2.9994990	-4.1937610
36	C	4.7054100	1.7555450	-3.4495140
37	H	4.7680570	1.0425170	-4.2664000
38	C	4.6233690	3.2897480	3.9034570
39	H	5.1132680	3.5218950	4.8444120
40	C	-0.6326920	4.7776800	0.6582950
41	H	-1.3628960	4.9235490	1.4466610
42	C	6.3045280	1.0604300	0.3727440
43	H	6.4789600	1.9184340	1.0136210
44	C	1.2617810	4.3953880	-1.3583530
45	H	2.0046360	4.2579370	-2.1378500
46	C	4.0467730	-4.2101910	-1.0588550
47	H	4.5335470	-4.3916840	-0.1046540
48	C	2.3272990	-3.0470560	2.2045780
49	H	1.3558430	-2.8636070	1.7569280
50	C	2.7217310	-2.7838000	-2.4972850
51	H	2.1627200	-1.8652250	-2.6509990
52	C	6.9294500	-0.3448530	-1.4979270
53	H	7.6067050	-0.5788430	-2.3136420

54	C	3.4359430	-4.9577240	-3.2777880
55	H	3.4552420	-5.7203820	-4.0512710
56	C	4.0809520	-5.1820250	-2.0581450
57	H	4.6043160	-6.1179660	-1.8830080
58	C	4.8224670	-3.4957750	3.3795310
59	H	5.7935560	-3.6666450	3.8364140
60	C	2.7573250	-3.7570270	-3.4965300
61	H	2.2399810	-3.5828440	-4.4356150
62	C	-0.5919200	5.6465910	-0.4337620
63	H	-1.3003110	6.4671490	-0.5000470
64	C	0.3482310	5.4476330	-1.4445730
65	H	0.3779770	6.1147140	-2.3016860
66	C	2.4175970	-3.7407310	3.4105220
67	H	1.5094840	-4.0966170	3.8874050
68	C	3.6619430	-3.9633950	4.0012130
69	H	3.7290960	-4.4963950	4.9457850
70	I	0.0550520	-0.0188370	2.1097210
71	Cu	-1.6805880	-0.0312830	0.0916940
72	P	-2.4871480	-2.2280650	0.0696100
73	P	-3.3120230	1.7051890	-0.0452900
74	O	-4.1526360	-0.4473950	-1.6703720
75	C	-3.6502690	-2.4817990	1.4579540
76	C	-1.1869960	-3.4911050	0.3358890
77	C	-4.5208520	-3.5930720	1.4499260
78	H	-4.4041850	-4.3588270	0.6870600
79	C	-3.2148870	-2.6753420	-1.4819510
80	C	-5.1221120	-0.2444030	-0.7176210
81	C	-4.9310700	0.8845470	0.0985750
82	C	-4.0360990	-1.7485260	-2.1966380
83	C	-3.3728760	2.6354060	-1.6163830
84	C	-3.3020830	-4.0468040	-1.9314630
85	H	-2.7418220	-4.8130240	-1.4050780
86	C	-3.8526540	-1.4735810	2.4189510
87	H	-3.1988450	-0.6053540	2.4268110
88	C	-5.5382510	-3.6967070	2.3905100
89	H	-6.1942800	-4.5636090	2.3772290
90	C	-0.2819830	-3.7752780	-0.6983280
91	H	-0.3337870	-3.2084730	-1.6235400
92	C	-5.8972020	1.2045140	1.0585350
93	H	-5.7521950	2.0744480	1.6922490
94	C	-4.0252380	-4.3793080	-3.0624590
95	H	-4.0289450	-5.4159150	-3.3930010
96	C	-3.4025980	3.0051180	1.2411370
97	C	-4.7466620	-2.0766950	-3.3220190
98	H	-5.3330710	-1.3038420	-3.8106070
99	C	-7.1710230	-0.7452780	0.4354340
100	H	-8.0261350	-1.3987750	0.5826010
101	C	-4.5749140	2.8608250	-2.2996970
102	H	-5.5040070	2.4645230	-1.8987340
103	C	-5.7239710	-2.6922420	3.3507850
104	H	-6.5266620	-2.7726430	4.0783350
105	C	-4.8779850	-1.5804640	3.3549160
106	H	-5.0188300	-0.7903270	4.0881010
107	C	-4.7280200	-3.4131890	-3.8074670
108	H	-5.2770250	-3.6815900	-4.7025500
109	C	0.6437760	-4.8073830	-0.5583180
110	H	1.3239750	-5.0320840	-1.3731970

111	C	-6.2344820	-1.0680820	-0.5446250
112	H	-6.3432300	-1.9528890	-1.1617730
113	C	-1.1228250	-4.2308090	1.5253360
114	H	-1.8228940	-4.0164050	2.3271230
115	C	-4.0246200	4.2400590	1.0121120
116	H	-4.4540660	4.4520290	0.0369030
117	C	-2.1732040	3.1289930	-2.1435160
118	H	-1.2369160	2.9402560	-1.6280870
119	C	-2.8378140	2.7418040	2.4961320
120	H	-2.3349240	1.7944510	2.6702570
121	C	-7.0191170	0.3948860	1.2270540
122	H	-7.7576010	0.6408900	1.9839990
123	C	-3.5080290	4.9320260	3.2728310
124	H	-3.5437520	5.6821220	4.0580940
125	C	-4.0767000	5.1985070	2.0240700
126	H	-4.5576920	6.1548680	1.8375470
127	C	-4.5750940	3.5784140	-3.4956530
128	H	-5.5096470	3.7441850	-4.0245180
129	C	-2.8903970	3.7013590	3.5079290
130	H	-2.4405690	3.4899950	4.4739790
131	C	0.6911370	-5.5527690	0.6216960
132	H	1.4174360	-6.3535630	0.7296080
133	C	-0.1907050	-5.2599570	1.6630330
134	H	-0.1599590	-5.8382910	2.5827300
135	C	-2.1784180	3.8499410	-3.3362630
136	H	-1.2407910	4.2232630	-3.7373170
137	C	-3.3772190	4.0742640	-4.0150180
138	H	-3.3784630	4.6265970	-4.9508780

Table S6. The atomic coordinates of the excited triplet state of CuIDPO in the computational results.

Tag	Element	*x*	*y*	*z*
1	I	-0.1069770	-0.0569530	-2.0814000
2	Cu	1.6286610	-0.0445060	-0.0633730
3	P	2.4416780	2.1661180	-0.0583130
4	P	3.2994730	-1.6859020	-0.0052880
5	O	4.2856370	0.4628180	1.6040480
6	C	3.6835540	2.4688080	-1.3715370
7	C	1.2316940	3.5258160	-0.2599360
8	C	4.5521720	3.5693170	-1.3319990
9	H	4.4980660	4.2686100	-0.5016350
10	C	3.3284350	2.6709510	1.4761370
11	C	5.1905020	0.2533680	0.5806570
12	C	4.9123380	-0.8487570	-0.2421960
13	C	4.1437820	1.7343390	2.1314020
14	C	3.4879690	-2.5783620	1.5775180
15	C	3.1974290	3.9390880	2.0608580
16	H	2.5689790	4.6823620	1.5804910
17	C	3.7668120	1.5631070	-2.4353450
18	H	3.0958130	0.7081490	-2.4586350
19	C	5.4871140	3.7605400	-2.3463150
20	H	6.1582270	4.6144620	-2.3098350
21	C	0.2581350	3.7092000	0.7361960
22	H	0.2118840	3.0270880	1.5808310
23	C	5.8068630	-1.1438720	-1.2763350
24	H	5.6077220	-1.9922630	-1.9238960

25	C	3.8413170	4.2488510	3.2592480
26	H	3.7217840	5.2372350	3.6932390
27	C	3.3658930	-3.0039740	-1.2732900
28	C	4.7767720	2.0247760	3.3357570
29	H	5.3745140	1.2528000	3.8100450
30	C	7.1695440	0.7585070	-0.6799470
31	H	8.0315500	1.3946030	-0.8580740
32	C	4.7376100	-2.8059530	2.1706550
33	H	5.6395510	-2.4378180	1.6884540
34	C	5.5651880	2.8526130	-3.4061110
35	H	6.2990310	2.9996350	-4.1938720
36	C	4.7053480	1.7554960	-3.4495520
37	H	4.7678010	1.0426910	-4.2667650
38	C	4.6233070	3.2896990	3.9034190
39	H	5.1136320	3.5219760	4.8442020
40	C	-0.6327540	4.7776290	0.6582570
41	H	-1.3622460	4.9245660	1.4471360
42	C	6.3044670	1.0603810	0.3727060
43	H	6.4780790	1.9188690	1.0131280
44	C	1.2617190	4.3953380	-1.3583910
45	H	2.0052300	4.2577870	-2.1372360
46	C	4.0467130	-4.2102400	-1.0588930
47	H	4.5346950	-4.3910490	-0.1051270
48	C	2.3272400	-3.0471050	2.2045400
49	H	1.3560170	-2.8580200	1.7588050
50	C	2.7216710	-2.7838500	-2.4973230
51	H	2.1622570	-1.8647790	-2.6496730
52	C	6.9293890	-0.3449010	-1.4979650
53	H	7.6063610	-0.5784480	-2.3141090
54	C	3.4358830	-4.9577740	-3.2778260
55	H	3.4562570	-5.7201710	-4.0516780
56	C	4.0808930	-5.1820740	-2.0581830
57	H	4.6054310	-6.1176000	-1.8837620
58	C	4.8224080	-3.4958230	3.3794930
59	H	5.7933910	-3.6663570	3.8368430
60	C	2.7572650	-3.7570760	-3.4965680
61	H	2.2408680	-3.5822080	-4.4361320
62	C	-0.5919830	5.6465400	-0.4338000
63	H	-1.2995400	6.4680190	-0.4995610
64	C	0.3481690	5.4475830	-1.4446110
65	H	0.3779620	6.1152530	-2.3014260
66	C	2.4175370	-3.7407810	3.4104840
67	H	1.5094810	-4.0948120	3.8891190
68	C	3.6618840	-3.9634440	4.0011750
69	H	3.7290190	-4.4960360	4.9460430
70	I	0.0549910	-0.0188870	2.1096830
71	Cu	-1.6806490	-0.0313340	0.0916560
72	P	-2.4872080	-2.2281160	0.0695720
73	P	-3.3120840	1.7051380	-0.0453280
74	O	-4.1526970	-0.4474460	-1.6704100
75	C	-3.6503290	-2.4818500	1.4579160
76	C	-1.1870560	-3.4911560	0.3358510
77	C	-4.5209120	-3.5931240	1.4498880
78	H	-4.3992920	-4.3646560	0.6939500
79	C	-3.2149470	-2.6753930	-1.4819890
80	C	-5.1221730	-0.2444550	-0.7176590
81	C	-4.9311310	0.8844950	0.0985370

82	C	-4.0361590	-1.7485780	-2.1966760
83	C	-3.3729370	2.6353550	-1.6164210
84	C	-3.3021430	-4.0468560	-1.9315010
85	H	-2.7435090	-4.8121940	-1.4017060
86	C	-3.8527140	-1.4736320	2.4189130
87	H	-3.1951230	-0.6074210	2.4277870
88	C	-5.5383110	-3.6967590	2.3904720
89	H	-6.1904470	-4.5665640	2.3792720
90	C	-0.2820430	-3.7753290	-0.6983660
91	H	-0.3240600	-3.1951480	-1.6165930
92	C	-5.8972630	1.2044620	1.0584970
93	H	-5.7520540	2.0752020	1.6909930
94	C	-4.0252980	-4.3793600	-3.0624970
95	H	-4.0380050	-5.4178710	-3.3860310
96	C	-3.4026600	3.0050670	1.2410990
97	C	-4.7467220	-2.0767460	-3.3220570
98	H	-5.3308770	-1.3030240	-3.8120100
99	C	-7.1710840	-0.7453310	0.4353960
100	H	-8.0266140	-1.3983670	0.5819680
101	C	-4.5749760	2.8607740	-2.2997350
102	H	-5.5047170	2.4661280	-1.8983250
103	C	-5.7240310	-2.6922940	3.3507470
104	H	-6.5265120	-2.7732700	4.0784360
105	C	-4.8780450	-1.5805150	3.3548780
106	H	-5.0164210	-0.7915990	4.0897380
107	C	-4.7280800	-3.4132400	-3.8075050
108	H	-5.2714840	-3.6802960	-4.7068850
109	C	0.6437160	-4.8074330	-0.5583560
110	H	1.3283630	-5.0277890	-1.3705900
111	C	-6.2345430	-1.0681340	-0.5446630
112	H	-6.3454860	-1.9533930	-1.1610900
113	C	-1.1228850	-4.2308590	1.5252980
114	H	-1.8203630	-4.0130160	2.3284170
115	C	-4.0246820	4.2400070	1.0120740
116	H	-4.4526050	4.4528530	0.0363750
117	C	-2.1732660	3.1289420	-2.1435540
118	H	-1.2361120	2.9380750	-1.6302970
119	C	-2.8378750	2.7417520	2.4960940
120	H	-2.3312900	1.7957640	2.6689820
121	C	-7.0191770	0.3948330	1.2270160
122	H	-7.7582840	0.6410050	1.9832590
123	C	-3.5080910	4.9319750	3.2727930
124	H	-3.5433360	5.6823490	4.0578170
125	C	-4.0767630	5.1984550	2.0240320
126	H	-4.5571660	6.1549820	1.8369740
127	C	-4.5751560	3.5783630	-3.4956910
128	H	-5.5100130	3.7450200	-4.0238800
129	C	-2.8904590	3.7013080	3.5078910
130	H	-2.4391920	3.4901890	4.4732790
131	C	0.6910780	-5.5528190	0.6216580
132	H	1.4208210	-6.3501200	0.7313300
133	C	-0.1907640	-5.2600070	1.6629950
134	H	-0.1592720	-5.8373130	2.5831780
135	C	-2.1784800	3.8498900	-3.3363010
136	H	-1.2404650	4.2224310	-3.7368480
137	C	-3.3772810	4.0742130	-4.0150560
138	H	-3.3786710	4.6269740	-4.9506830

Table S7. The atomic coordinates of CO_2, benzene and CO_2 + benzene in the computational results.

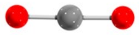

CO_2

Tag	Element	*x*	*y*	*z*
1	O	1.1679610	0.1173050	0.0000000
2	O	-1.1679610	-0.1315560	0.0000000
3	C	0.0000000	0.0190010	0.0000000

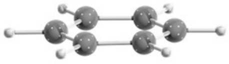

Benzene

Tag	Element	*x*	*y*	*z*
1	C	0.7782930	1.1419840	-0.0142930
2	H	1.3851280	2.0498550	-0.0211770
3	C	1.3911540	-0.1059710	0.0149100
4	C	-0.6042230	1.2380930	-0.0019700
5	H	-1.0765590	2.2224230	-0.0012420
6	C	-1.3800320	0.1021560	0.0149450
7	H	-2.4690660	0.1851950	0.0283730
8	C	0.5978090	-1.2383040	-0.0007360
9	H	1.0649930	-2.2254130	-0.0051760
10	C	-0.7816700	-1.1378020	-0.0134490
11	H	-1.3916940	-2.0429500	-0.0245750
12	H	2.4792130	-0.1900510	0.0273550

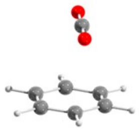

CO_2+Benzene

Tag	Element	*x*	*y*	*z*
1	O	1.8533240	-1.4425100	-0.2303970
2	O	2.8285210	0.6769350	0.0442100
3	C	2.3329390	-0.3853870	-0.0683530
4	C	-0.8178720	0.5773850	1.3111340
5	H	-0.4960200	0.8851850	2.3082280
6	C	-1.5253400	-0.6078080	1.1418680
7	C	-0.5473210	1.3875500	0.2197260
8	H	-0.0118700	2.3278040	0.3654120
9	C	-0.9544170	1.0151970	-1.0404900
10	H	-0.7411460	1.6602790	-1.8957430
11	C	-1.8994370	-0.9838090	-0.1350400
12	H	-2.4320610	-1.9250270	-0.2869810
13	C	-1.6055950	-0.1841230	-1.2247110
14	H	-1.9099740	-0.4969030	-2.2252440
15	H	-1.7614330	-1.2407680	1.9990250

Table S8. The atomic coordinates of CH_3CN, benzene and CH_3CN + benzene in the computational results.

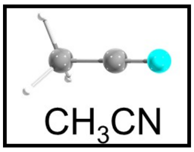

Tag	Element	*x*	*y*	*z*
1	H	-1.5478060	0.9987520	-0.2598130
2	H	-1.5566640	-0.2722260	0.9928310
3	N	1.4391850	0.0005980	-0.0006650
4	C	0.2722750	-0.0021430	0.0016640
5	C	-1.1744360	0.0008660	-0.0009380
6	H	-1.5568570	-0.7230540	-0.7327160

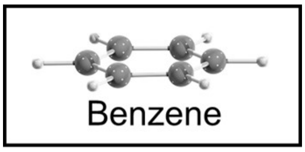

Tag	Element	*x*	*y*	*z*
1	C	0.7556210	-1.1592090	0.0101080
2	H	1.3449600	-2.0779670	0.0245730
3	C	1.3927820	0.0723910	-0.0077970
4	C	-0.6295400	-1.2309840	-0.0050550
5	H	-1.1228040	-2.2035960	0.0075850
6	C	0.6267530	1.2369120	-0.0025390
7	H	1.1146420	2.2132930	-0.0017220
8	C	-1.3855720	-0.0720500	-0.0117320
9	H	-2.4749380	-0.1317520	-0.0020710
10	C	-0.7587150	1.1529180	0.0105690
11	H	-1.3525340	2.0692260	0.0242380
12	H	2.4826950	0.1309230	-0.0139330

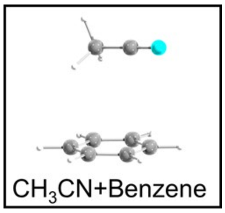

Tag	Element	*x*	*y*	*z*
1	H	-0.8384600	1.3599200	-0.1330480
2	H	-2.2140720	1.9415020	0.8437000
3	C	1.1495630	-0.1540100	1.4010000
4	H	1.0698150	-0.1557240	2.4897080
5	C	1.5437640	0.9978840	0.7370140
6	C	0.8694120	-1.3111290	0.6892120
7	H	0.5615490	-2.2132370	1.2191810
8	C	1.6183810	0.9913230	-0.6548660
9	H	1.9130190	1.8948400	-1.1917130
10	N	-2.8981250	-1.0331770	-0.0047880
11	C	0.9563650	-1.3159010	-0.6917930
12	H	0.7146960	-2.2215830	-1.2501050
13	C	1.3086120	-0.1645180	-1.3583000
14	H	1.3599970	-0.1598190	-2.4490640
15	C	-2.4699860	0.0520460	-0.0308510

16	C	-1.9323810	1.3946720	-0.0672780
17	H	-2.3239840	1.9397580	-0.9363770
18	H	1.7819300	1.9043820	1.2964010

3. Reference

[1] H. Yersin, A. F. Rausch, R. Czerwieniec, T. Hofbeck, T. Fischer. The triplet state of organo-transition metal compounds. Triplet harvesting and singlet harvesting for efficient OLEDs. *Coord. Chem. Rev*. **2011**, 255, 2622

[2] M. J. Frisch, G. W. Trucks, H. B. Schlegel, G. E. Scuseria, M. A. Robb, J. R. Cheeseman, G. Scalmani, V. Barone, B. Mennucci, G. A. Petersson, H. Nakatsuji, M. Caricato, X. Li, H. P. Hratchian, A. F. Izmaylov, J. Bloino, G. Zheng, J. L. Sonnenberg, M. Hada, M. Ehara, K. Toyota, R. Fukuda, J. Hasegawa, M. Ishida, T. Nakajima, Y. Honda, O. Kitao, H. Nakai, T. Vreven, Jr J. A. Montgomery, J. E. Peralta, F. Ogliaro, M. Bearpark, J. J. Heyd, E. Brothers, K. N. Kudin, V. N. Staroverov, R. Kobayashi, J. Normand, K. Raghavachari, A. Rendell, J. C. Burant, S. S. Iyengar, J. Tomasi, M. Cossi, N. Rega, J. M. Millam, M. Klene, J. E. Knox, J. B. Cross, V. Bakken, C. Adamo, J. Jaramillo, R. Gomperts, R. E. Stratmann, O. Yazyev, A. J. Austin, R. Cammi, C. Pomelli, J. W. Ochterski, R. L. Martin, K. Morokuma, V. G. Zakrzewski, G. A. Voth, P. Salvador, J. J. Dannenberg, S. Dapprich, A. D. Daniels, O. Farkas, J. B. Foresman, J. V. Ortiz, J. Cioslowski, D. J. Fox. *Gaussian, Inc.*, Wallingford CT, **2009**.

[3] C. Adamo, V. Barone. Toward reliable density functional methods without adjustable parameters: The PBE0 model. *J. Chem. Phys.* **1999**, 110, 6158.

[4] T. Lu, F. Chen. *J. Comput. Chem*. **2012**, 33, 580.

[5] Humphrey, W.; Dalke, A.; Schulten, K., VMD: Visual Molecular Dynamics. *J. Mol. Graphics Modell*. **1996**, 14, 33-38.

[6] J. P. Perdew, K. Burke and M. Ernzerhof. Generalized Gradient Approximation Made Simple. *Phys. Rev. Lett.* **1996**, 77, 3865-3868.

[7] S. Grimme. Semiempirical GGA-type density functional constructed with a long-range dispersion correction. *J. Comput. Chem*. **2006**, 27, 1787-1799.
